# PAR2 regulates regeneration, transdifferentiation, and death

**DOI:** 10.1038/cddis.2016.357

**Published:** 2016-11-03

**Authors:** Ron Piran, Seung-Hee Lee, Pia Kuss, Ergeng Hao, Robbin Newlin, José Luis Millán, Fred Levine

**Affiliations:** 1 Sanford Children's Health Research Center, Sanford Burnham Prebys Medical Discovery Institute, La Jolla, CA, USA

## Abstract

Understanding the mechanisms by which cells sense and respond to injury is central to developing therapies to enhance tissue regeneration. Previously, we showed that pancreatic injury consisting of acinar cell damage+*β*-cell ablation led to islet cell transdifferentiation. Here, we report that the molecular mechanism for this requires activating protease-activated receptor-2 (PAR2), a G-protein-coupled receptor. PAR2 modulation was sufficient to induce islet cell transdifferentiation in the absence of *β*-cells. Its expression was modulated in an islet cell type-specific manner in murine and human type 1 diabetes (T1D). In addition to transdifferentiation, PAR2 regulated *β*-cell apoptosis in pancreatitis. PAR2's role in regeneration is broad, as mice lacking PAR2 had marked phenotypes in response to injury in the liver and in digit regeneration following amputation. These studies provide a pharmacologically relevant target to induce tissue regeneration in a number of diseases, including T1D.

The ability of a tissue to regenerate after injury is fundamental to organismal survival, but the mechanisms by which cells sense and respond to injury remain poorly understood.^1^ Cell and tissue damage, particularly to pancreatic *β*-cells, is a fundamental aspect of diabetes, being caused by *β*-cell autoimmunity in type 1 diabetes (T1D) and by obesity-associated factors in type 2 diabetes (T2D). Therefore, promoting *β*-cell regeneration has been a major focus of diabetes research.

Recently, we found that acinar cell damage induced by pancreatic duct ligation (PDL) in the absence of *β*-cells led to islet cell transdifferentiation by transdifferentiation of *α*-cells to *β*-cells.^2, 3^ Surgical reversal of PDL led to recovery from severe diabetes,^4^ demonstrating translational potential. We then replaced the complex surgical procedure of PDL with caerulein, a pharmacologically relevant intervention,^5^ which induces acinar cell hypersecretion and is used to model pancreatitis.^6, 7^ Caerulein plus *β*-cell ablation induced *α*- to *β*-cell transdifferentiation, with some *β*-cells further transdifferentiating into *δ*-cells expressing somatostatin.^5^ Evidence of ongoing transdifferentiation was found in T1D, both in the NOD mouse and human patients, ultimately leading to increased *δ*-cell number, analogous to what occurred with caerulein.^5^

Having demonstrated islet transdifferentiation in two distinct pancreatitis models, we reasoned that a factor common to pancreatitis in general must be having an important role. One feature common to all forms of pancreatitis is the release of pancreatic exocrine enzymes. Among those are proteases. Thus, we identified protease-activated receptors (PARs) as candidates for stimulating islet cell transdifferentiation.

PARs are G-protein-coupled receptors (GPCRs) that participate in diverse processes, including thrombosis, pain, and inflammation.^8, 9, 10, 11, 12, 13^ There are four PARs, of which the best candidate for inducing islet cell transdifferentiation was protease-activated receptor-2 (PAR2), as it is activated by trypsin,^14, 15, 16, 17, 18^ which cleaves a fragment from the its N-terminus,^8, 9, 10, 11, 12, 13^ leading to internalization and degradation,^9, 10^ resulting in decreased PAR2 levels.^19^

PAR2 is being investigated as a therapeutic target, with pain and inflammation as primary indications.^20, 21^ Therefore, many modulators of PAR2 have been developed.^20, 22^ Generally, PAR2 agonists lead to PAR2 internalization and degradation, whereas antagonists inhibit internalization.^22, 23^

Here, we used mice carrying a mutated, inactivated PAR2 (PAR2 knockout (PAR2KO)),^8^ and pharmacological activator to show that PAR2 was necessary and sufficient for islet cell transdifferentiation induction in the setting of *β*-cell deficiency. Surprisingly, we found that PAR2 was required for *β*-cell survival during caerulein-mediated pancreatitis. Furthermore, it was important in the injury response in other organs, including the liver and extremities. The ability of a pharmacologically relevant single target to control regeneration and survival in multiple tissues identifies PAR2 as a therapeutic target in those and possibly other disorders involving tissue damage and regeneration, including T1D.

## Results

### A PAR2 modulator induced islet cell transdifferentiation in *β*-cell ablated mice

To begin to test the hypothesis that PAR2 partakes in islet cell transdifferentiation induced by pancreatitis+*β*-cell ablation, islet PAR2 expression was examined. Human islet RNA-seq data^24^ revealed that, of the four PARs, PAR2 was highly expressed, with PAR1 being present and PAR3 and PAR4 essentially undetectable (Supplementary Figure S1A). Next, we examined the effect of the PAR2 agonist, 2-furoyl-LIGRLO-amide trifluoroacetate salt (2fLI).^25^

**Figure 1:**
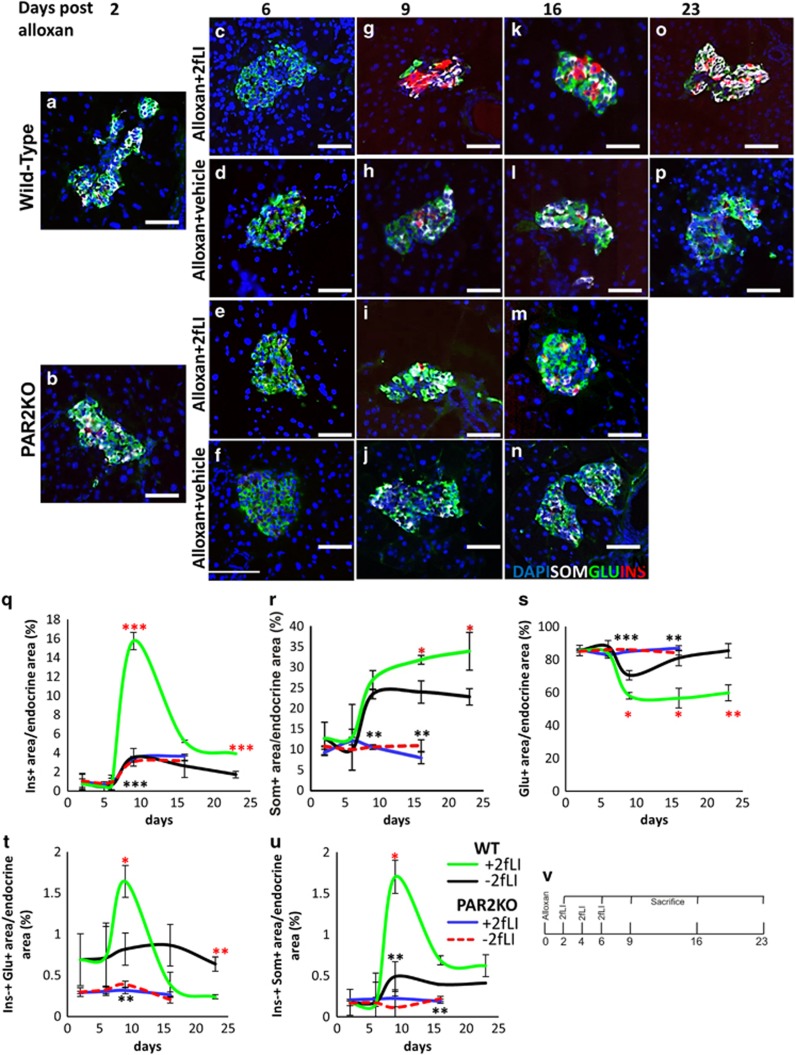
PAR2 was sufficient to induce islet cell transdifferentiation in *β*-cell ablated mice. *β*-Cell depleted islets from WT (**a**) and PAR2KO (**b**) mice 2 days after alloxan injection. 2fLI or vehicle were injected into WT or PAR2KO mice on days 2, 4, and 6. Mice were killed on days 6 (**c-f**), 9 (**g-j**), 16 (**k-n**), and 23 (**o** and **p**). Pancreas sections were analyzed for insulin (red), glucagon (green), and somatostatin (white). Alloxan injected PAR2KO mice did not survive to day 23 (Supplementary Figure S2L), and did not respond to 2fLI (**e**, **f**, **i**, **j**, **m**, **n**, quantified in **q-u**). (**q**) Quantification of insulin-positive area over time, demonstrating a peak of *β*-cells at day 9 following alloxan+2fLI (green line), with no significant change following alloxan+vehicle (black line). Alloxan+2fLI treated PAR2KO mice were not responsive (blue line). (**r**) Quantification of somatostatin-positive area over time, demonstrating continued increase of *δ*-cell formation following alloxan+2fLI (green line), with little change following alloxan+vehicle (black line). Alloxan+2fLI treated PAR2 mice were not responsive (blue line). (**s**) Quantification of glucagon-positive area over time, demonstrating decrease in the number of *α*-cells following alloxan+2fLI (green line), with little change following alloxan+vehicle (black line). Alloxan+2fLI treated PAR2 mice were not responsive (blue line). (**t**) Quantification of insulin-glucagon co-positive area, demonstrating an increase of these cells following alloxan+2fLI peaking at day 9 (green line), and declining at day 23 when such cells were no longer present. (**u**) Quantification of insulin-somatostatin co-positive area, demonstrating an increase of coexpressing cells following alloxan+2fLI, peaking at day 9 and declining by day 23 (green line). Quantification was performed using an Aperio digital imaging system (Materials and methods section). Endocrine cell size was not significantly changed as a result of the 2fLI treatment (8.26*10^−3^±3.53*10^−3^*μ*m^2^ for alloxan alone and 8.10*10^−3^±3.79*10^−3^*μ*m^2^ for alloxan+2fLI). Note that the key for panels (**q**–**u**) is shown in (**u**). (**v**) Schematic of experimental design. Error bars are S.E.M. Red asterisk is between WT alloxan+2fLI and WT alloxan+vehicle. Black asterisk is between PAR2KO alloxan+2fLI and WT alloxan+2fLI. Scale bar= 75 *μ*m

Mice were depleted of their *β*-cells by alloxan (Figure 1a, additional examples in Supplementary Figure S2A), followed by 2fLI injections on days 2, 4, and 6 (schematic in Figure 1v). 2fLI induced a marked increase in the number of cells expressing insulin (compare Figures 1g and k quantified in 1q with Figures 1h and l, additional examples in Supplementary Figures S2E and F *versus* S2B and C). If those cells arose by transdifferentiation from *α*-cells, intermediates coexpressing insulin and glucagon should exist. Such cells were present (compare Figures 1g and k with Figures 1h and l, quantified in 1 t, additional examples in Supplementary Figures S2E and F *versus* S2B and C. High-power magnifications of insulin-glucagon co-positive cells are in S3A and B). Later, there was an increase in cells expressing somatostatin (compare Figures 1k and o, with 1l and p, quantified in 1r, additional examples in Supplementary Figures S2G *versus* S2D), concomitantly with insulin-somatostatin coexpressing cells (Figure 1u, high-power magnification of an insulin-somatostatin co-positive cell in S3C and D), indicating that *δ*-cells formed from *β*-cells.^5^ This accounted for the *β*-cell decrease at day 16 (Figure 1q). To rule out the possibility that changes in endocrine cell sub-populations could occur by replication or apoptosis, we checked for Ki67, a proliferation marker, and cleaved caspase 3, an apoptosis marker, in islets, but found no evidence of those markers in islets from mice treated with alloxan+2fLI (Supplementary Figure S4), indicating that neither proliferation nor apoptosis are significant factors in this process.

Consistent with our previous results,^5^ no transdifferentiation was observed in the presence of *β*-cells, and alloxan+vehicle was ineffective at inducing transdifferentiation (Figures 1d, h, l and p, quantified in 1q–u, additional examples in Supplementary Figures S2B–D). Although the mice were not followed for a long period, which we found previously to be required for changes in blood glucose to occur, there was a marked survival advantage in the alloxan+2fLI group (Supplementary Figure S2L).

**Figure 2:**
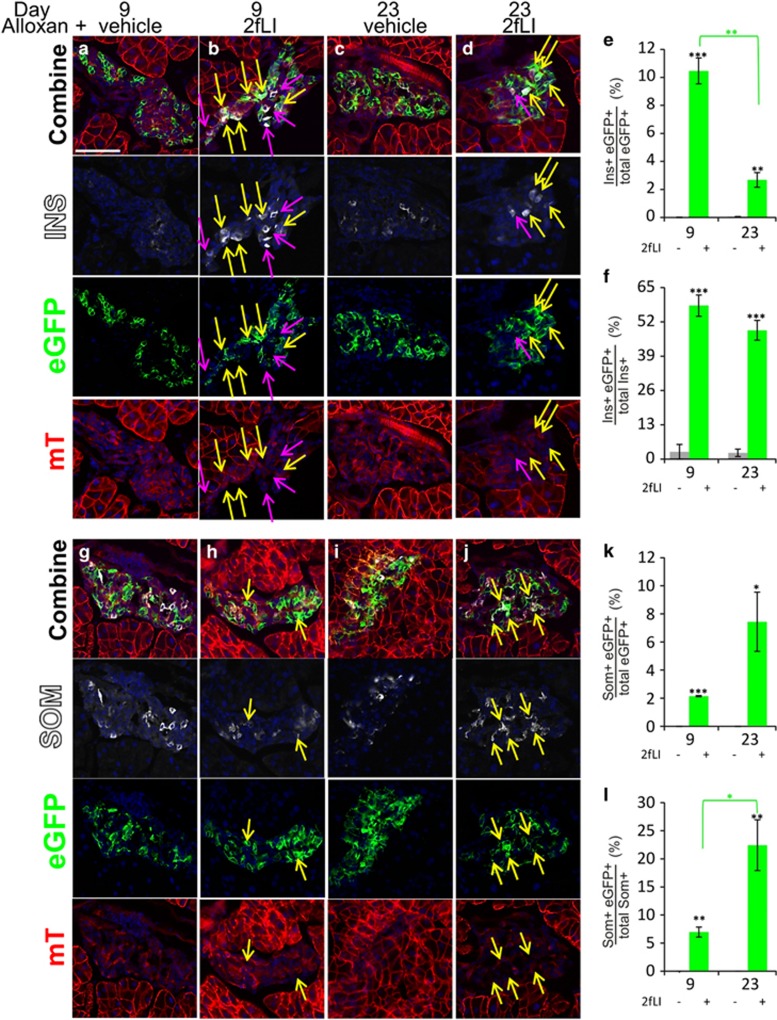
Genetic lineage tracing demonstrated transdifferentiation of *α*- to *β*- and *δ*-cells following alloxan+2fLI. Glu-mT/mG mice were injected with alloxan+2fLI as described in Figure 1. (**a**–**d**) Representative islets from mice killed at days 9 (**a** and **b**) and 23 (**c** and **d**), treated with alloxan+vehicle (**a** and **c**) or with alloxan+2fLI (**b** and **d**) and stained for insulin (white). High-power views of cells within islets (**b** and **d**) are in Supplementary Figure S7. (**e** and **f**) Quantification of transdifferentiated *β*-cells (insulin+ eGFP+) as a fraction of total eGFP+ cells (**e**), or total insulin-positive cells (**f**). (**g-j**) Representative islets from mice killed at days 9 (**g** and **h**) and 23 (**i** and **j**), treated with alloxan+vehicle (**g** and **i**) or alloxan+2fLI (**h** and **j**) and stained for somatostatin. High-power views of cells within islets (**h** and **j**) are in Supplementary Figure S8. (**k** and **l**) Quantification of transdifferentiated *δ*-cells (somatostatin+ eGFP+) as a percentage of total eGFP+ cells (**k**), or total somatostatin+ cells (**l**). Note that no eGFP+ cells expressing somatostatin were detected after alloxan+vehicle. Error bars are S.E.M. Scale bar=75 *μ*m

For definitive proof of islet cell transdifferentiation induced by 2fLI, we used genetic lineage tracing of *α*-cells by crossing mice expressing a glucagon promoter-cre transgene with mice expressing cell membrane-localized mT in their tissues before Cre recombinase exposure, and eGFP after cre-mediated recombination (Glu-mT/mG) as described previously^5^ (Figure 2). No eGFP-positive cells were detected in the islets of those mice before mating (Supplementary Figure S5). In the crossed mice, 60.1% of *α-*cells were eGFP+ (Supplementary Figure S6). Following alloxan+vehicle treatment, eGFP-insulin co-positive cells were extremely rare (<0.02% at day 9, and ~0.4% at day 23, Figures 2a and c, quantified in e). In contrast, insulin-positive cells expressing membrane-localized eGFP, indicating an origin from recombined *α*-cells, were found at a high frequency following alloxan+2fLI (Figures 2b and d, quantified in 2e and f, high-power views in S7). At day 9, 10.5% of all eGFP+ cells were insulin positive, and this was 58.2% of all *β*-cells. All eGFP+ cells were in islets. None were ever found in ducts. Thus, 2fLI stimulated a marked increase in *α*- to *β*-cell transdifferentiation.

As with alloxan+caerulein,^5^ we observed an increase over time in somatostatin-eGFP co-positive cells, indicating an origin from recombined *α*-cells, with a corresponding decrease in insulin-expressing cells. Somatostatin-eGFP co-positive cells rose from 2.1% of total eGFP+ cells at day 9 to 7.4% at day 23 (Figures 2h and j; quantification in Figures 2e and f for the decrease in insulin-eGFP cells and Figures 2k and l for the increase in somatostatin-eGFP cells; high-power views are in S8.). No cells coexpressing somatostatin and eGFP were ever found in mice treated with alloxan+vehicle (Figures 2g, i, k and l). As we have never found cells coexpressing glucagon and somatostatin, either in this or in our previous study,^5^ the transdifferentiation process from *α*- to *δ*-cells most likely proceeds through a *β*-cell intermediate, although it is formally possible, albeit unlikely, that if direct *α*- to *δ*-cell transdifferentiation invariably occurred in *α*-cells that had lost glucagon expression, then *δ*-cells could have arisen directly from *α*-cells.

As we did previously,^5^ we took advantage of variability in the extent of *β*-cell ablation following alloxan varied from islet to islet to measure the degree to which transdifferentiation occurred in islets that differed in the number of surviving *β*-cells. Using lineage marking to distinguish between preexisting *β*-cells and neogenic *β*-cells, we counted the number of preexisting (mT-expressing) and neogenic (eGFP-expressing) *β*-cells in each islet. In islets with efficient *β*-cell ablation, there were many neogenic *β*-cells (Supplementary Figures S9A-C), whereas in islets with many surviving *β*-cells, there was little to no transdifferentiation, that is, mT-expressing *β*-cells (Supplementary Figure S9D-F). There was a threshold effect, with the vast majority of transdifferentiated *β*-cells found in islets with seven or fewer preexisting *β*-cells (Supplementary Figure S9G, *P*<2e-10), which is in substantial agreement with our previous observation.^5^ This effect is consistent with a local sensing mechanism where each islet behaves autonomously with respect to *β*-cell neogenesis.

### Transdifferentiation induced by 2fLI required PAR2

To prove that the ability of 2fLI to stimulate islet cell transdifferentiation was through PAR2, we performed experiments with mice in which the *F2RL1* gene, encoding PAR2, was mutated, rendering it inactive.^8^ In those mice (PAR2KO), which are viable and without significant phenotype developmentally or as adults under normal conditions,^8^ being bred as homozygotes, alloxan+2fLI had no effect on transdifferentiation, with no change in the frequency of cells expressing single hormones, and few to no cells expressing multiple hormones (Figures 1b, e, i and m, quantified in 1q–u, additional examples in Supplementary Figures S2J and K). Thus, PAR2 is required for islet cell transdifferentiation mediated by 2fLI.

### PAR2 activation stimulated *α*- to *β*-cell transdifferentiation but no proliferation

In both the caerulein and PDL models, islet cells replicated in addition to transdifferentiating, although replication and transdifferentiation were independent of one another.^3, 5^ To determine whether PAR2 activation affected proliferation, an independent group of mice was treated with alloxan+2fLI along with continuous BrdU administration to label replicating cells. As *β*-cell transdifferentiation reached its peak about 2 weeks following alloxan+PDL or caerulein, mice were killed on days 9 and 16 to examine for islet cell neogenesis and replication.^3, 5^ As observed earlier (Figure 1), alloxan+2fLI induced an increase in the number of insulin and somatostatin expressing cells (compare Supplementary Figure S10A with S10B). However, in contrast to caerulein and PDL, no proliferation was seen following alloxan+2fLI as evidenced by no BrdU-positive cells, although such cells were apparent in the intestine (Supplementary Figure S10C), a highly replicative organ. This is consistent with the decrease in the number of *α*-cells following alloxan+2fLI, reflecting depletion of the pool of *α*-cells as they transdifferentiated. Thus, although PDL- and caerulein-induced pancreatitis led to transdifferentiation and proliferation, PAR2 modulation led only to transdifferentiation.

**Figure 3:**
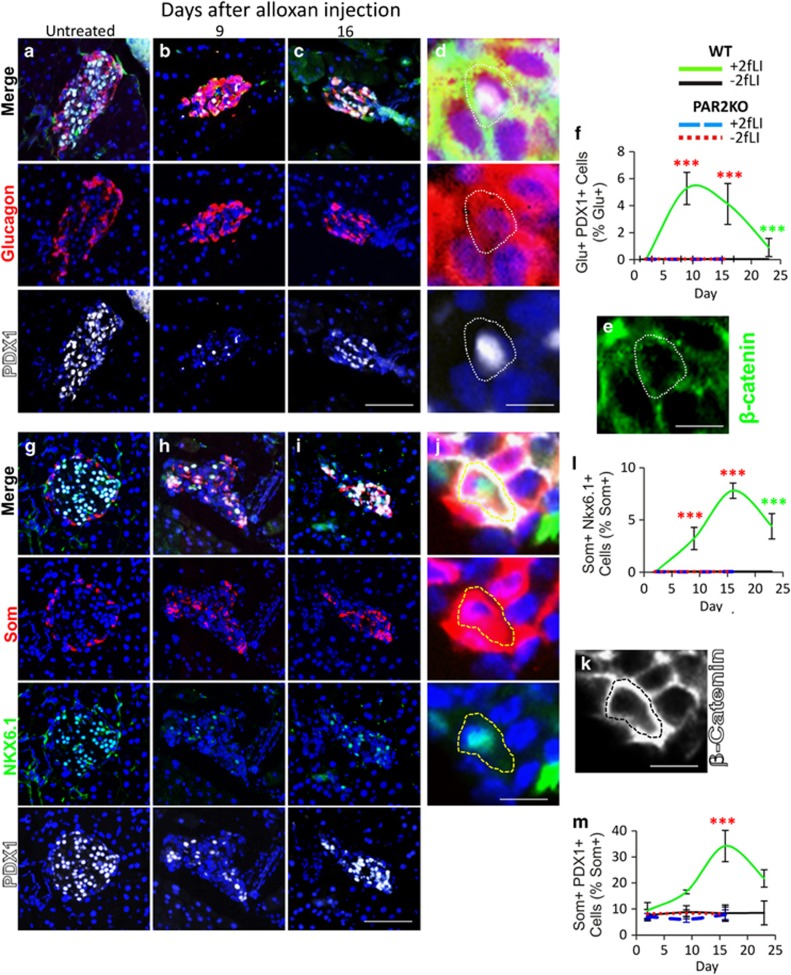
Transitional cells expressing PDX1 and NKX6.1 were induced by 2fLI. (**a–d**) Cells coexpressing PDX1 (white) and glucagon (red) were not found in untreated mice (**a**), but were found following 2fLI at days 9 (**b**, **d** and **e**) and 16 (**c**). (**d** and **e**) A high-power view of a cell coexpressing PDX1 and glucagon, in which *β*-catenin (green in **e**) was used to outline cells to confirm marker coexpression. (quantification in **f**). Cells coexpressing somatostatin (red) and NKX6.1 (green) or PDX1 (white) were not found in untreated mice (**g**) but increased following alloxan plus 2fLI at day 9 (**h**) and day 16 (**i** and **j**), but not after alloxan alone. (**j** and **k**) A high-power view of a cell coexpressing NKX6.1 and somatostatin, in which *β*-catenin (white in **k**) was used to outline cells to confirm marker coexpression. (**l** and **m**) Quantification of cells coexpressing somatostatin and Nkx6.1 or PDX1, respectively. Scale bar for lower power views= 75 *μ*m, high-power views=6.7 *μ*m

As we showed previously in the alloxan+caerulein model,^5^ *α*- to *β*-cell transdifferentiation was accompanied by the occurrence of cells coexpressing glucagon and transcription factors found in *β*-cells. PDX1 and NKX6.1 are expressed in all *β*-cells, with NKX6.1 being highly *β*-cell specific^26, 27^ (Figures 3a and g, quantified in l, whereas PDX1 is also expressed in some *δ*-cells ^28^(Figure 3g, quantified in f and m). Following alloxan+2fLI, there was an increase in PDX1-glucagon coexpressing cells, reaching a peak at day 9 when 5.3% of glucagon-positive cells also expressed PDX1 (Figures 3a and e, quantified in f). As recently reported, such cells were not found in untreated or *β*-cell ablated mice ^5^ and their occurrence is most consistent with *α*- to *β*-cell direct conversion, rather than with a model in which *α*-cells dedifferentiate before transdifferentiation. As predicted by the presence of *β*- to *δ*-cell transdifferentiation, and as found in the caerulein model,^5^ we found somatostatin-NKX6.1 coexpressing cells (Figures 3g and k, quantified in l). Such cells were not found in controls – either alloxan+vehicle treated wild-type (WT) mice or PAR2KO mice (Figure 3l). In addition to somatostatin-NKX6.1 coexpressing cells, the percentage of somatostatin-PDX1 coexpressing cells rose following alloxn+2fLI (Figures 3g and k, quantified in m). No cells coexpressing glucagon and PDX1 or Nkx6.1, or somatostatin and Nkx6.1, were found in alloxan+vehicle treated WT or PAR2KO mice (Figures 3f and l).

### PAR2 expression in the exocrine and endocrine pancreas was altered in pancreatitis and T1D

**Figure 4:**
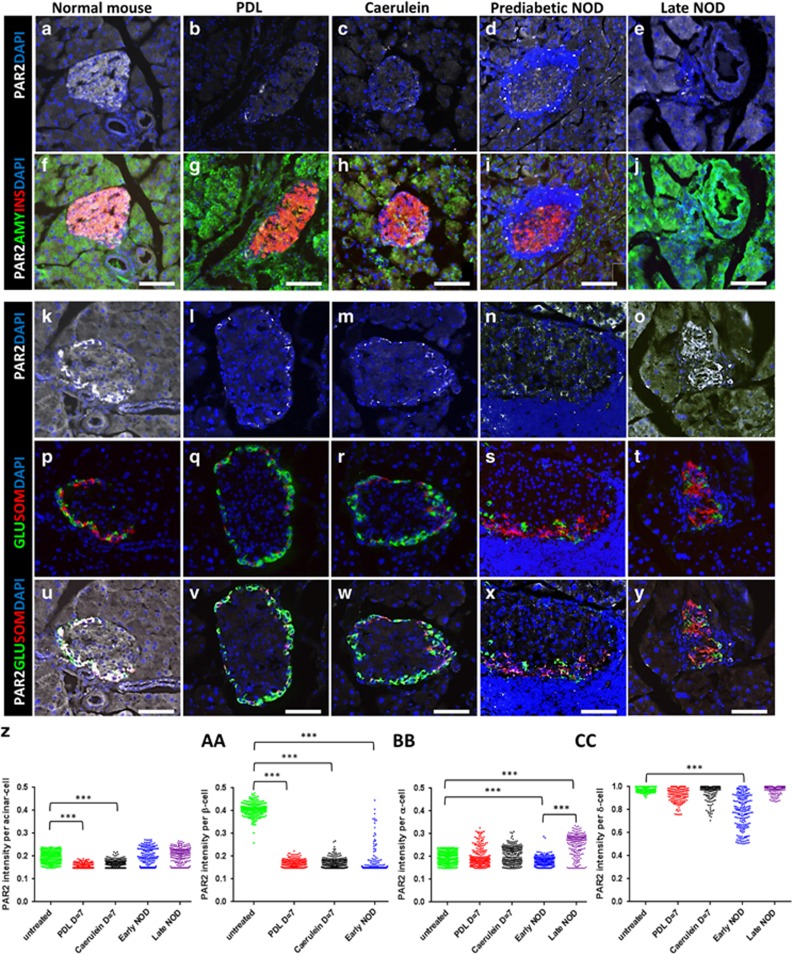
PAR2 expression in pancreatitis and T1D. The five columns show immunostaining of pancreas sections from a normal mouse (**a**, **f**, **k**, **p** and **u**), a mouse that had undergone PDL (**b**, **g**, **l**, **q** and **v**) a mouse injected with caerulein (**c**, **h**, **m**, **r** and **w**), a prediabetic NOD mouse with insulitis (**d**, **i**, **n**, **s** and **x**), and a late stage diabetic NOD mouse (**e**, **j**, **o**, **t** and **y**). Sections were stained for PAR2 (white in **a–o** and **u-y**), amylase (green in **f-j**), insulin (red in **f-j**), glucagon (green in **p-y**), and somatostatin (red in **p-y**). DAPI was used in all panels to visualize nuclei. Note that for each condition, two separate islets are shown in the upper two *versus* lower three panels. (**z**, **aa**, **bb** and **cc**) Quantification of PAR2 expression in the acinar, *β*-, *α*-, and *δ*-cells, respectively. In each condition, *P*-values were calculated by Kruskal–Wallis test in comparison with untreated mice. Scale bars=75 *μ*m

**Figure 5:**
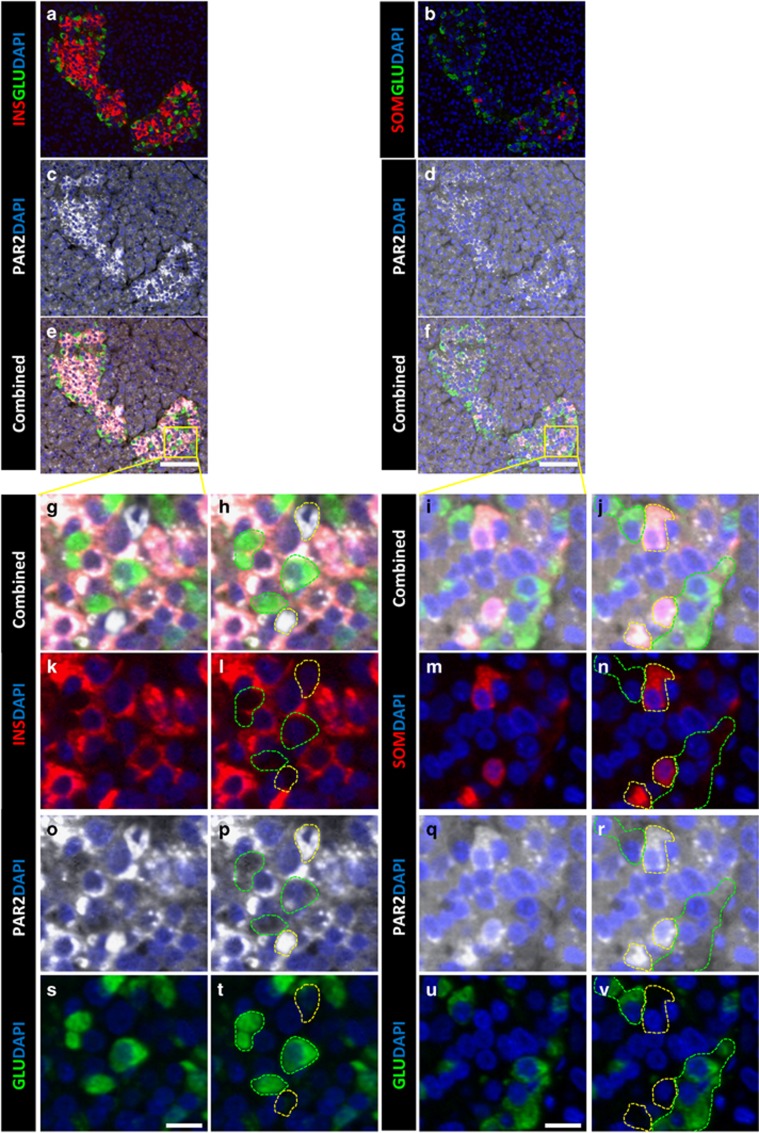
PAR2 expression in the human endocrine pancreas. Serial sections (left *versus* right columns) from a human pancreas stained for PAR2 (white in **c-j** and **o–r**), glucagon (green in **a**, **b**, **e-j** and **s-v**), insulin (red in **a**, **e**, **g**, **h**, **k** and **l**) and somatostatin (red in **b**, **f**, **i**, **j**, **m** and **n**). As in the mouse (Figures 4 and 6), the expression of PAR2 in the islet cells was highest in *δ*-cells (surrounded by yellow dashed line in **h**, **j**, **l**, **n**, **p**, **r**, **t**, **v**), intermediate in *β*-cells, and lowest in *α*-cells (surrounded by green dashed line in **h**, **j**, **l**, **n**, **p**, **r**, **t** and **v**). (**a-f**) show low power views (scale bars=75 *μ*m), whereas (**g-v**) show high-power views of the areas in the yellow squares (scale bars=10 *μ*m)

Having shown that 2fLI induced islet cell transdifferentiation, it was interesting to determine the pattern of PAR2 expression in the pancreas and islets. PAR2 was expressed throughout the pancreas, but was higher in the islets of mice (Figures 4a, f, k and u) and humans (Figure 5, PAR2 antibody validation is in Supplementary Figures S11A–D). Within the islets of both mice and humans, the highest PAR2 level was in δ-cells (Figures 4k, p and u, quantified in cc, 5b, d, f, i, j, m, n, q and r. High-power views in Supplementary Figures S11E–G). *β*-Cells had a lower level (Figures 4a and f, quantified in aa, 5a, c, e, g, h, k, l, o and p). *α*-Cells had the same level of expression as in the surrounding acinar tissue (Figures 4a, f, k, p and u, quantified in z, bb, 5e–j and s–v).

Following PDL and caerulein, PAR2 expression was greatly reduced in *β*-cells (Figures 4b, c, g and h, quantified in aa). In *α*-cells, PAR2 increased, but only in a sub-population (Figures 4l, m, q, r, v and w, quantified in bb). PAR2 expression in *δ*-cells decreased, (Figures 4l, m, q, r, v and w, quantified in cc).

In T1D, PAR2 was reduced in the *β*-cells of prediabetic NOD mouse islets that had substantial insulitis (Figures 4d and i). However, islets without insulitis retained high PAR2 expression (Supplementary Figure S12), indicating that local effects of insulitis were responsible for the decrease. *α*-Cells and *δ*-cells exhibited distinctive patterns of PAR2 expression, with *α*-cell PAR2 expression going down initially but then rising to higher levels than baseline, whereas *δ*-cell PAR2 expression decreased initially but then returned to baseline in late stage NOD mice (Figures 4n, s, x, o, t and y, quantified in bb and cc, respectively).

### 2fLI induced the same pattern of changes in PAR2 expression as occurred in pancreatitis and T1D

**Figure 6:**
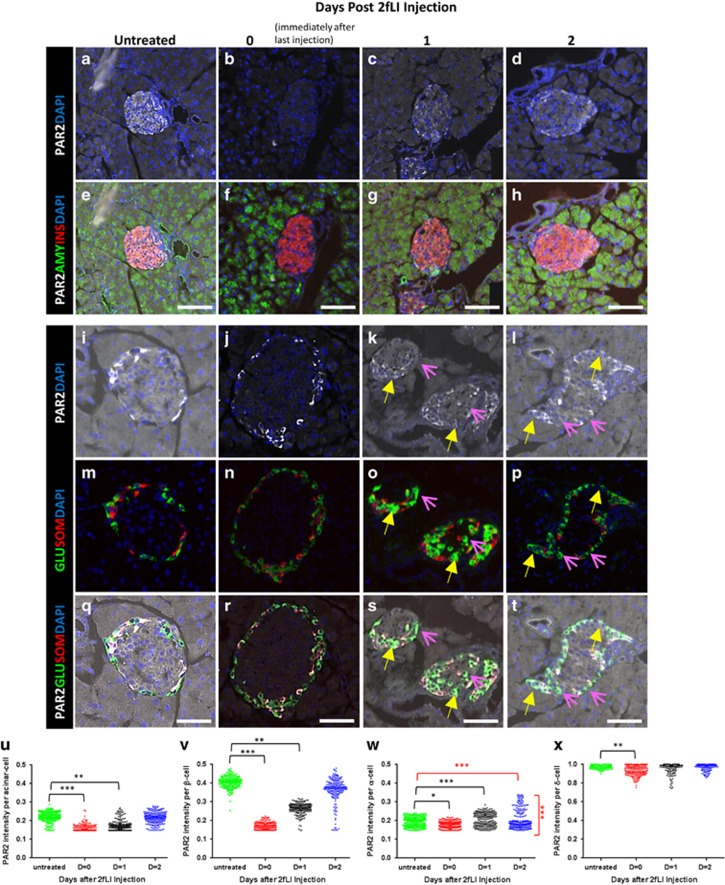
PAR2 was differentially expressed and modulated by 2fLI in the exocrine and endocrine pancreas. Pancreas sections stained for PAR2 (white in **a–l** and **q-t**), amylase (green in **e-h**), insulin (red in **e-h**), glucagon (green in **m–t**), and somatostatin (red in **m–t**). DAPI (blue) was used to visualize nuclei. For each condition, two separate islets are shown in the upper two *versus* lower three panels. Staining for the indicated markers from untreated mice (**a**, **e**, **i**, **m** and **q**. Note that these controls were also used for Figure 4) or at the indicated times following 2fLI last injection. At time 0 (**b**, **f**, **j**, **n** and **r**), PAR2 was reduced by 2fLI in all cell types. PAR2 expression returned to baseline over time – 1  day (**c**, **g**, **k**, **o** and **s**) and 2 days (**d**, **h**, **l**, **p** and **t**, quantified in **u-x**), except in *α*-cells, where a sub-population exhibited increased PAR2 expression (yellow arrows). High-power views of the areas indicated areas in (**k**, **l**, **o**, **p**, **s** and **t**) are in Supplementary Figures S14 and S15. (**u-x**) PAR2 expression. Quantification in the acinar, *β*-, *α*-, and *δ*-cells. *P*-values shown in black were calculated by Kruskal–Wallis one-way analysis of variance test in comparison with untreated mice. The *α*-cell population was bimodal (*P*-value <0.005 by Mann–Whitney test – shown in red). Both the high and low PAR2 *α*-cell sub-populations were significantly different from *α*-cells from untreated mice (*P*<0.005 for both groups in red). Scale bars=75 *μ*m

If PAR2 was the principal or only effector in the induction of islet cell transdifferentiation that occurred in pancreatitis and T1D, then 2fLI should elicit the same changes in PAR2 expression as were seen in those disease states. Consistent with 2fLI acting directly on the islet cells, and similarly to pancreatitis and T1D, 2fLI affected PAR2 expression in the pancreas, with differential effects on islet cells. In *β*-cells, 2fLI induced substantial downregulation of PAR2, whereas there was a small decrease in PAR2 in acinar cells (Figures 6a–h, quantified in u and v). The most interesting finding was that 2fLI induced changes in PAR2 expression in *α*-cells that were similar to what occurred in pancreatitis, where PAR2 expression increased in a sub-population (Figures 6i and t, quantified in w). This led to a bimodal distribution of PAR2 expression in *α*-cells, with one population being significantly lower (pink arrows in Figures 6k, l, o, p, s and t) relative to a sub-population where the PAR2 level was significantly higher than the control (yellow arrows in Figures 6k, l, o, p, s and t. High-power views of the islets shown in Figures 6q, s, and t are in Supplementary Figures S13–S15, respectively). *δ*-Cells expressed a high level of PAR2 at baseline and this decreased slightly in response to 2fLI, similar to pancreatitis and T1D (Figures 6i–t, quantified in x). Note that the increased PAR2 expression in a sub-population of *α*-cells occurred when *β*-cells were present, where no transdifferentiation occurred (Figure 1).^3, 5^ This suggests that increased PAR2 expression in *α*-cells is an early event that is independent of the effects of *β*-cell deficiency, consistent with the two factor model of islet cell transdifferentiation that we put forth previously.^5^

### *α*-Cell to *β*-cell transdifferentiation occurred in glucagon-positive cells expressing a high level of PAR2

**Figure 7:**
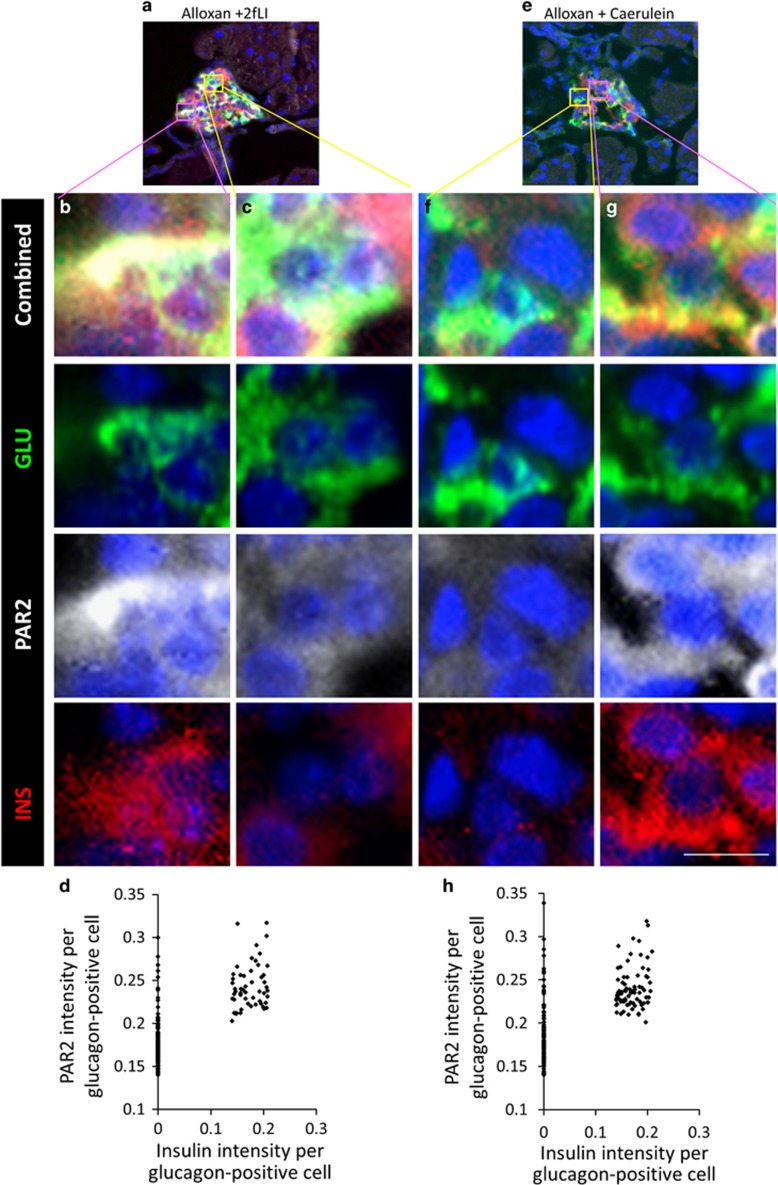
2fLI and caerulein-induced PAR2 elevation in a sub-population of *α*-cells. *β*-Cell ablated mice were injected with 2fLI (**a-c**) or caerulein (**e-g**) and the pancreases harvested for analysis of insulin (red), glucagon (green), and PAR2 (white). Both 2fLI and caerulein-induced elevated PAR2 expression in a sub-population of *α*-cells in *β*-cell ablated mice, the majority of which coexpressed insulin. (**a** and **e**) Low-power views of islets from *β*-cell ablated mice injected with 2fLI (**a**) or caerulein (**e**) mice. (**b** and **g**) High-power views of cells expressing glucagon, a high level of PAR2, and insulin. (**c** and **f**) High-power views of cells expressing glucagon, a low level of PAR2, and no insulin. (**d** and **h**) Quantification of PAR2 and insulin expression in cells that expressed glucagon from mice injected with 2fLI (**d**) or caerulein (**h**). In *β*-cell ablated mice injected with 2fLI, cells expressing glucagon but not insulin had a significantly lower level of PAR2 than those cells from mice not injected with 2fLI (*P*=0.0051). Cells coexpressing glucagon and insulin in *β*-cell ablated mice injected with 2fLI or caerulein had a significantly higher level of PAR2 than cells expressing only glucagon (defined as having an arbitrary PAR2 intensity >0.21 *P*<2.2e-16). Scale bar=7  *μ*m

**Figure 8:**
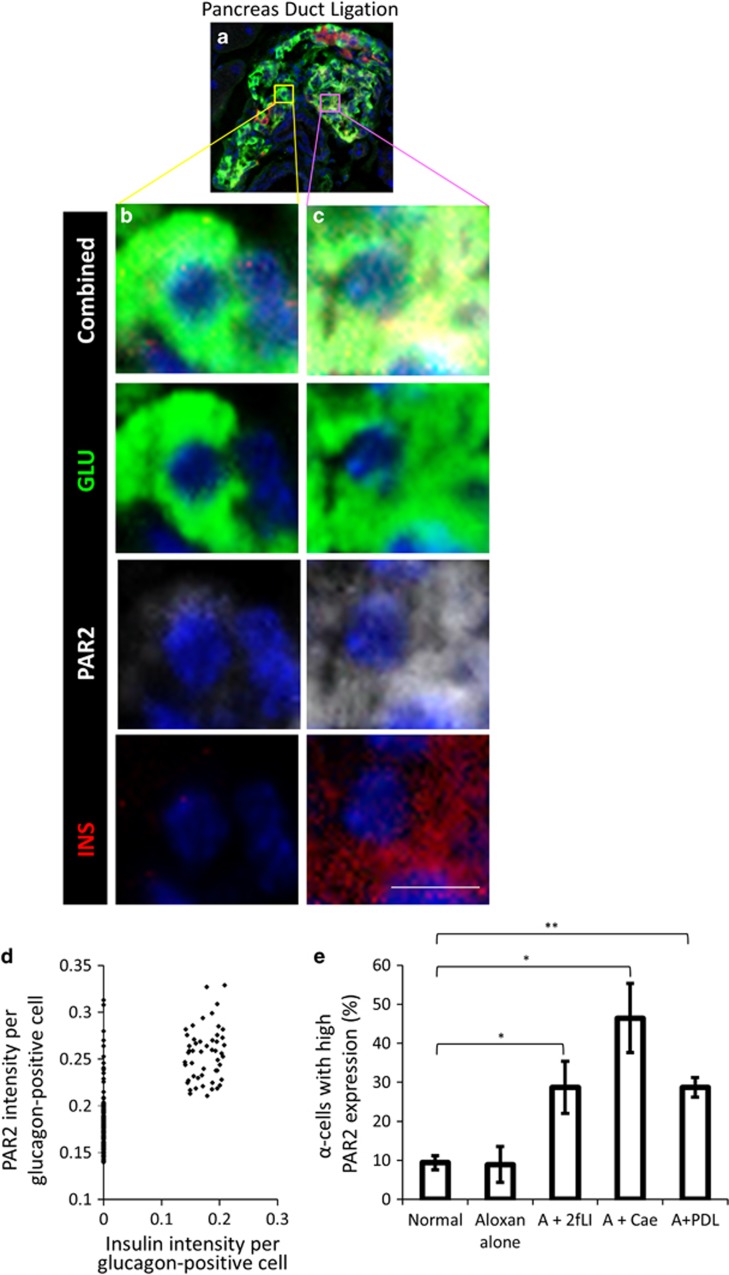
*β*-Cell ablation combined with PDL induced PAR2 elevation in sub-population of *α*-cells. In *β*-cell ablated mice subjected to PDL (**a**), cells coexpressing glucagon and insulin exhibited high PAR2 expression (**c**) relative to cells expressing only glucagon (**b**, quantified in **d**). Cells coexpressing glucagon and insulin in *β*-cell ablated mice subjected to PDL had a significantly higher level of PAR2 than cells expressing only glucagon (*P*<2.2e-16). (**e**) Quantification of the percentage of cells expressing high PAR2 (defined as having an arbitrary PAR2 intensity >0.21) in normal mice, *β*-cell ablated mice, *β*-cell ablated mice injected with 2fLI, caerulein, or subjected to PDL. Scale bar=7 *μ*m

Normal (Supplementary Figures S16A-C, quantified in D) and *β*-cell ablated (Supplementary Figures S16E-G, quantified in H) mice had a small number of *α*-cells with high PAR2, but these cells did not coexpress insulin (Supplementary Figures S16D and H).There was no difference in the number of *α*-cells exhibiting high PAR2 expression after *β*-cell ablation alone (*P*=0.97). This is consistent with the *α*-cell lineage tracing that found only rare instances of islet cell transdifferentiation in untreated or alloxan+vehicle-treated mice (Figure 2). However, in *β*-cell ablated mice injected with 2fLI (Figures 7a–c, quantified in d), caerulein (Figures 7e–g, quantified in h), or subjected to PDL (Figures 8a and c, quantified in d), most glucagon-positive cells with high PAR2 expression coexpressed insulin.

**Figure 9:**
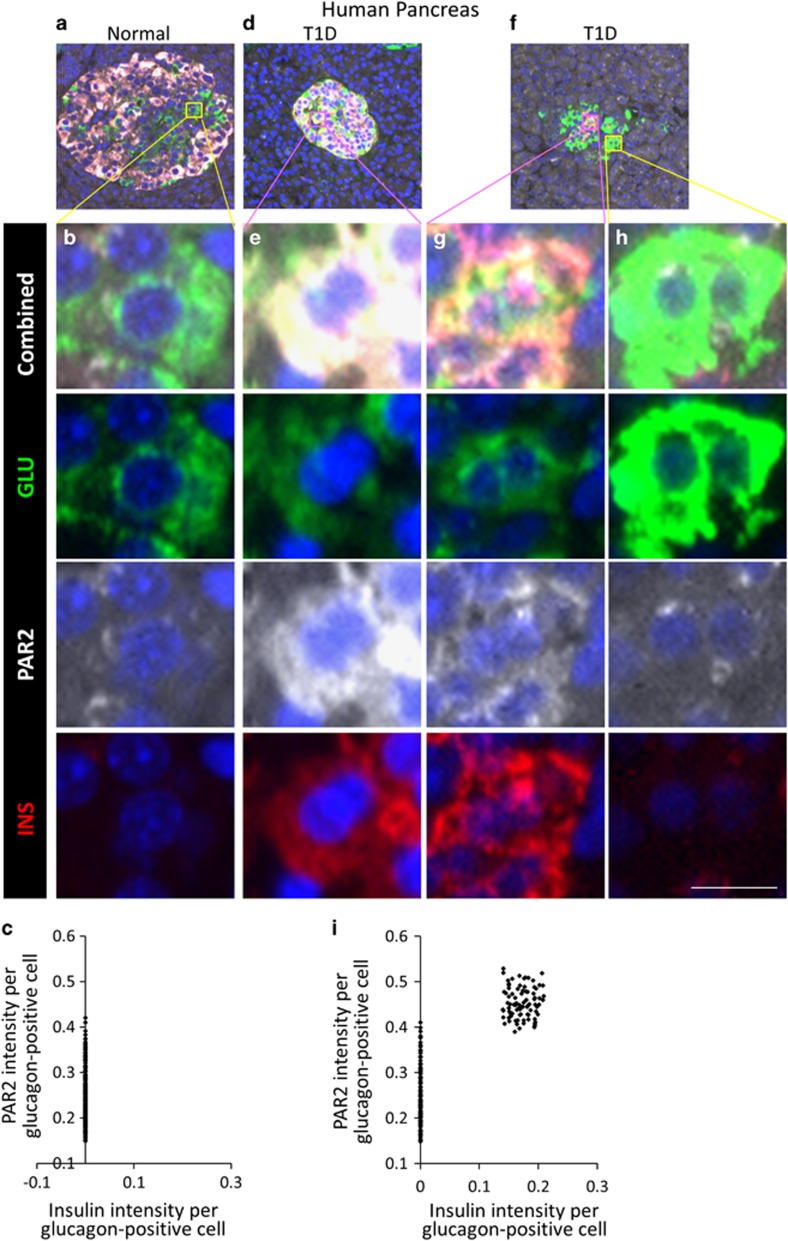
In human T1D, islet cells coexpressing glucagon and insulin exhibited high PAR2 expression. Non-diabetic nPOD donors (nPOD#6282 shown as an example in **a** and **b**) had no cells coexpressing glucagon (green) and insulin (red)(quantified in **c**). Some patients with T1D (nPOD donors 6083 and 6052 shown in (**d** and **f**), respectively, with corresponding high-power views in (**e** and **g**), high PAR2 levels and insulin-positive, compared with **h** low PAR2 levels and insulin negative) had cells coexpressing glucagon and insulin, and those cells had a higher level of PAR2 (white) expression (quantified in **i**). Low-power view of the islet shown in (**d**) is shown in Supplementary Figure S17 with the PAR2 channel separated to demonstrate more clearly the differential PAR2 expression. Cells coexpressing glucagon and insulin in T1D had a significantly higher level of PAR2 than cells expressing only glucagon (*P*<2.2e-16). Scale bar=7 *μ*m

To determine whether PAR2 exhibited the same changes in expression in human islet cells as occurred in the mouse, we examined sections from normal and T1D human pancreases. Similar to mice, *α*-cells in non-diabetic humans expressed a relatively low level of PAR2, and no glucagon-insulin co-positive cells were found (Figures 9a and b, quantified in c). As we reported previously,^5^ some patients with T1D had glucagon-insulin coexpressing cells (Figures 9d and g, Supplementary Figure S17). Although there was no difference in the level of PAR2 expression between *α*-cells (which were insulin negative) of normal (Figures 9a and b), and T1D patients (Figures 9f and h), all glucagon-insulin co-positive cells exhibited high PAR2 expression (quantified in Figure 9i), as predicted by the murine studies. In contrast to the mouse, where a small number of *α*-cells had high PAR2 expression, consistent with the very low but detectable level of transdifferentiation with *β*-cell ablation alone seen by us ^5^ and others;^29^ in normal or T1D patients there were no normal *α*-cells with high PAR2 expression, suggesting that PAR2 regulation is more stringent in humans than in mice. Given the heterogeneity of T1D in humans, it will be important to study more cases to fully understand the complexity of PAR2 expression in human islet cells.

### PAR2 controlled hormone gene expression through a PAX4-dependent pathway

**Figure 10:**
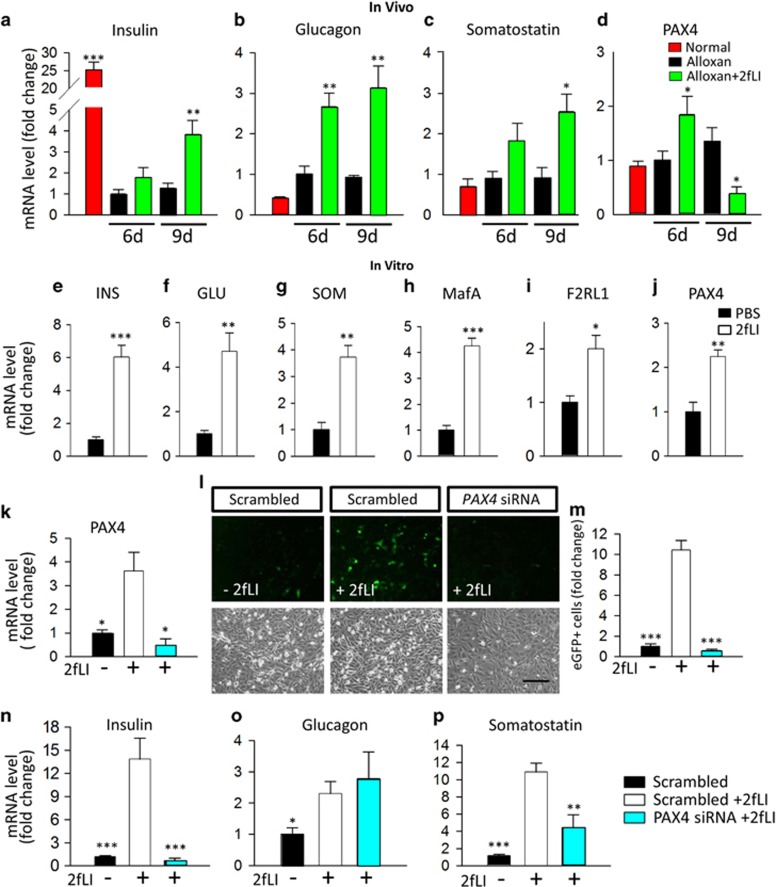
PAR2 regulated hormone genes *in vivo* and *in vitro* through a PAX4-dependent pathway. (**a–d**) *In vivo* studies: islets from normal control mice (red), alloxan+vehicle (black) and alloxan+2fLI (green) were purified 6 and 9 days after alloxan or vehicle injection and used for RNA isolation and quantitative RT-PCR for (**a**) insulin (**b**) glucagon (**c**) somatostatin and (**d**) PAX4. *sP*-values are relative to day 6 alloxan+vehicle. (**e-j**) *In vitro* studies. T6PNE cells were treated with 2fLI (10 *μ*M) for 4 days, and RNA was isolated for quantitative RT-PCR for (**e**) insulin, (**f**) glucagon, (**g**) somatostatin, (**h**) MafA, (**i**) F2RL1, and (**j**) PAX4. (**k-p**) PAX4 siRNA studies: in a different experiment from (**e**–**j**), T6PNE cells were transfected with scrambled siRNA or Pax4 siRNA and cultured for 5 days. Where indicated, vehicle or 2fLI (10 *μ*M) was added for the last 3 days before harvest. (**k**) PAX4, but not scrambled siRNA suppressed PAX4 mRNA, demonstrating efficacy of the PAX4 siRNA. (**l**) 2fLI induced the expression of eGFP that is driven in T6PNE cells by the human insulin promoter.^30^ eGFP was extinguished by PAX4, but not scrambled, siRNA. (**m**) Quantification of the number of cells expressing eGFP in the experiment shown in panel(**l**). (**n-p**) RT-PCR for insulin (**n**), Glucagon (**o**), and somatostatin (**p**), demonstrating dependence of insulin and somatostatin, but not glucagon gene expression on PAX4 downstream of PAR2. *P*-values are relative to scrambled siRNA+2fLI. Error bars are S.E.M. Scale bars=200 *μ*m

To study the mechanism by which islet cell transdifferentiation occurred *in vivo*, we performed quantitative RT-PCR analysis on RNA from *β*-cell ablated islets isolated from mice at two time points: immediately following the last injection vehicle or 2fLI (day 6) and 3 days after the last injection (day 9). Compared with vehicle, 2fLI induced increases in insulin, glucagon, and somatostatin expression levels (Figures 10a–c).

*In vitro* studies with the human islet cell line T6PNE ^30^ were performed to determine whether the effect of 2fLI on hormone gene expression was cell autonomous and whether effects on hormone expression were an intrinsic aspect of PAR2 activation. Similar to the *in vivo* effect, 2fLI induced an increase in mRNA of all three major hormones (Figures 10e and g). It also induced the insulin transactivator MafA (Figure 10h), and the PAR2 gene *F2RL1* itself (Figure 10i). The *F2RL1* induction by 2fLI indicates the existence of a positive autoregulatory feedback loop controlling PAR2 gene expression. Interestingly, the 2fLI effect on insulin expression *in vitro* was mimicked by *F2RL1* siRNA. As expected, both 2fLI and *F2RL1* siRNA led to decreased PAR2 expression (Supplementary Figures S18G-J, quantified in K and L), as 2fLI induces PAR2 protein internalization and degradation, whereas *F2RL1* siRNA inhibits expression at the mRNA level. Similar to 2fLI (Supplementary Figures S18A and B, quantified in E), *F2RL1* siRNA increased human insulin promoter activity (Supplementary Figures S18C and D, quantified in F), suggesting that loss of PAR2 from the cell surface, whether by ligand induced internalization or by an siRNA, determines the level of PAR2 signaling. This is consistent with the fact that many GPCRs exhibit substantial signaling in the basal state.^31^ However, additional work will be required to understand how PAR2 signaling induces transdifferentiation and insulin promoter activation.

Previous studies showed that the transcription factor PAX4 promotes *α*- to *β*-cell transdifferentiation, as ectopic expression of that gene in *α*-cells was sufficient to drive *β*-cell transdifferentiation.^32, 33^ Although Pax4 has been thought to be absent from adult islets as determined by immunohistochemistry ^34, 35^ or microarray analysis,^36^ RNA-seq of adult human islets revealed that it is present.^24, 37^ Strikingly, PAX4 expression was induced by 2fLI *in vivo* (Figure 10d) and *in vitro* (Figure 10j).

To determine whether the effect of 2fLI on hormone gene expression was dependent on PAX4, we used PAX4 siRNA, which successfully reduced the level of PAX4 mRNA (Figure 10k). It ablated the 2fLI effect on the human insulin promoter-eGFP transgene (Figures 10l and m) and endogenous human insulin gene (Figure 10n) *in vitro*. It also decreased the 2fLI effect on somatostatin gene expression (Figure 10p) but had no effect on glucagon expression (Figure 10o).

### PAR2 was required for pancreatic acinar regeneration following caerulein-induced injury

The fact that PAR2 was activated in caerulein-induced pancreatitis and key role that it had in recovery from injury following *β*-cell ablation begged the question of whether PAR2 had a role in the recovery from exocrine cell damage induced by caerulein. PAR2 protein level in the pancreas was greatly reduced by caerulein (Figures 4c, h, m and w), consistent with activation, followed by internalization and degradation.^14^ Furthermore, gene expression microarray data from the pancreases of caerulein-treated mice ^38^ revealed that *F2RL1* mRNA, encoding PAR2, was significantly increased (Supplementary Figure S1B), as predicted if the positive autoregulatory loop posited above (Figure 10i) was active.

**Figure 11:**
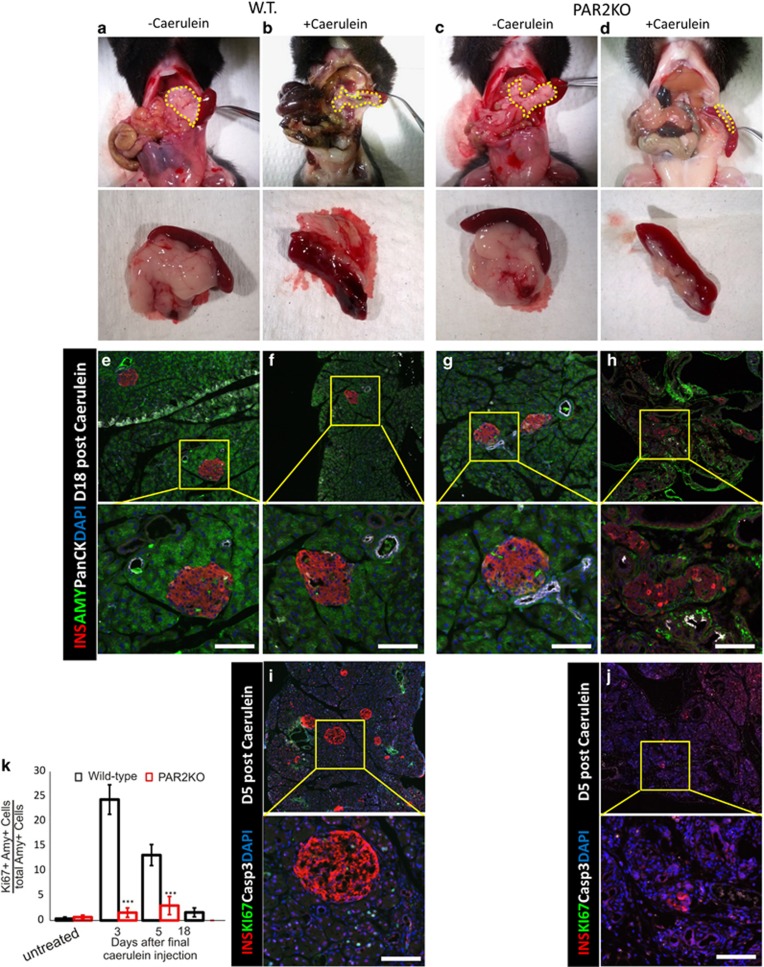
PAR2 was required for pancreas regeneration following caerulein injection. WT (**a** and **b**) and PAR2KO (**c** and **d**) mice injected with vehicle (**a** and **c**) or caerulein (**b** and **d**) were killed 18 days following injection. Macroscopic views of the abdominal cavity (upper panel) and harvested pancreas/spleen (lower panel) demonstrated severe pancreatic hypoplasia following caerulein injection in the PAR2KO mouse. Pancreases in upper panel are outlined with dashed yellow lines. (**e-h**) Microscopic views of samples corresponding to (**a–d**), respectively. Immunofluorescence for insulin (red), amylase (green) and pancytokeratin (white). Note that the acinar tissue of WT mice recovered completely from caerulein treatment, whereas the PAR2KO pancreas did not (compare amylase staining in **f** *versus* **h**). (**i** and **j**) Immunofluorescence for insulin (red), Ki67 (green), and cleaved caspase 3 (white) in caerulein-treated WT and PAR2KO pancreases, respectively. Ki67+ cells are apparent in the WT (**i**) but not in the PAR2KO (**j**). Nuclei are stained with DAPI (blue). Scale bars= 75 *μ*m. (**k**) Quantification of Ki67+ cells following caerulein injections. Error bars are S.E.M.

**Figure 12:**
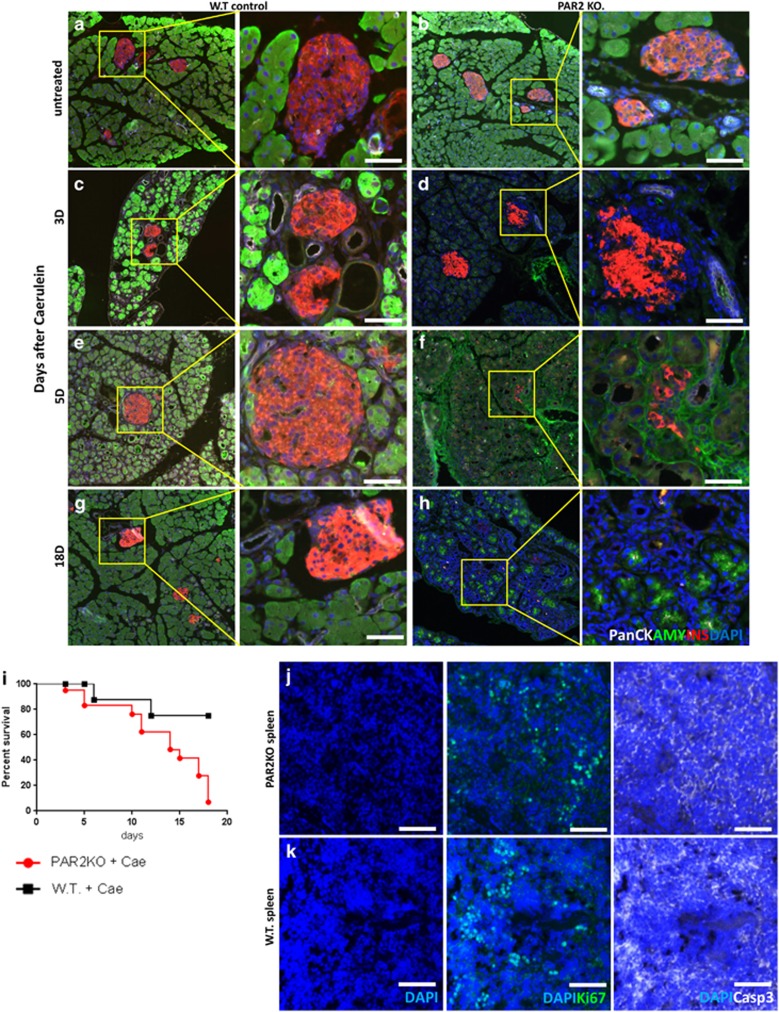
Pancreatic regeneration following caerulein injection. As in Figure 11, pancreas sections were stained for insulin (red), amylase (green), panCK (white), with nuclei visualized with DAPI (blue). A time course was performed before caerulein (**a** and **b**), and at day 3 (**c** and **d**), 5, (**e** and **f**), and 18 (**g** and **h**) following the last injection of caerulein. (**i**) Kaplan–Meier survival plot of WT and PAR2KO mice following caerulein injection. *P*-value for the survival difference between the WT (black) and PAR2KO mice (red) was 0.0048 by the log-rank (Mantel–Cox) test. (**j** and **k**) Spleens from WT and PAR2KO mice were stained for cleaved caspase 3 (white) and Ki67 (green) as a positive control. Scale bar=75 *μ*m

To determine directly whether PAR2 had a role in the recovery from caerulein-induced pancreatitis, caerulein was injected into the PAR2KO mouse. Although the pancreases of untreated WT and PAR2KO mice appeared the same (compare Figures 11a with c and e with g), there was a defect in pancreatic acinar regeneration in caerulein-treated PAR2KO mice, leading to severe pancreatic hypoplasia (compare Figures 11b with d and f with h, 12c, e and g with 12d, f and h). There was a highly significant negative effect of caerulein on survival in the PAR2KO (Figure 12i).

To examine possible mechanisms for the pancreatic hypoplasia following caerulein in the PAR2KO, we measured proliferation and apoptosis. In neither WT nor PAR2KO did we find acinar cells positive for cleaved caspase 3 (Figures 11i and j), although many such cells were seen in the spleen (Figures 12j and k). As expected,^5^ proliferation was increased in in the WT pancreas following caerulein as determined by Ki67 staining (Figure 11i), which was absent in the PAR2KO (Figure 11j, quantified in k), indicating a defect in the replicative response required for regeneration. Therefore, not only does PAR2 have a role in islet cell transdifferentiation, but it has a broader role in pancreatic regeneration.

### PAR2 was required for *β*-cell, but not *α*- or *δ*-cell, survival

**Figure 13:**
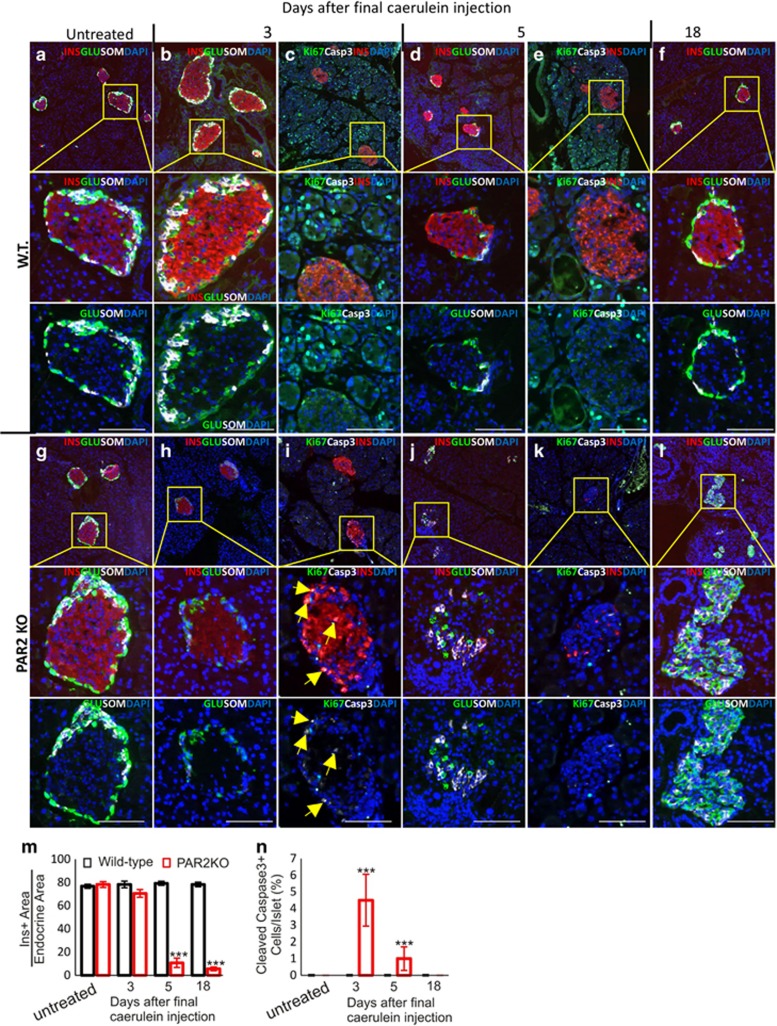
PAR2 has a protective role specific to *β*-cells in caerulein-induced pancreatitis. (**a**–**l**) Time course analysis of hormone expression, replication, and apoptosis. In WT pancreases (**a-f**), the acinar cells regenerated and exhibited robust replication, indicated by the many Ki67-positive cells (**c** and **e**), whereas the islet remained Ki67 negative, unaffected by caerulein-induced pancreatitis. In PAR2KO (**g-l**), *β*-cells were lost in response to caerulein-induced pancreatitis (quantified in **m**). Three days after the last caerulein injection, the central area of the islet usually occupied by *β*-cells was insulin-positive (red) but had no nuclei (absence of DAPI staining in **h-i**). Caerulein-induced *β*-cell death was mediated by apoptosis (cleaved caspase 3 marked by yellow arrows in **i**, quantified in **n**), but no increase in apoptosis was detected in the exocrine pancreas (**i** and **k**). This led to islets composed entirely of *α*- (green) and *δ*-cells (white) (**l**). Scale bars=75 *μ*m. (**m**) Insulin-positive area/endocrine area. (**n**) Number of cleaved caspase 3 positive cells/Islet. *P*-values are relative to the WT on the same day. Scale bars=75 *μ*m. Error bars are S.E.M.

In WT mice, caerulein had no effect on islets, (Figures 11f, i, and 13b–f). However, in caerulein-treated PAR2KO mice there was specific loss of *β*-cells (Figures 11h, j and 13h–l). Three days after the final caerulein injection, *β*-cells retained insulin immunoreactivity, but there were no intact nuclei (note absence of DAPI staining in areas of insulin positivity in Figures 13h and i). Ultimately, the islets consisted almost of *α*- and *δ*-cells, with essentially no *β*-cells (Figures 13j and l, quantified in m). In contrast with the acinar pancreas, where cleaved caspase 3 was not found, it was present in the caerulein-treated PAR2KO *β*-cells (Figures 13i and k, quantified in n). Thus, PAR2 is important in cell survival and proliferation within the pancreas, and specifically protects *β*-cell from apoptosis.

### PAR2 participated in tissue regeneration: CCl_4_-induced liver injury

The finding that PAR2 had a role in acinar cell regeneration in addition to *β*-cell transdifferentiation and survival raised the possibility that it has a general role in tissue regeneration. To test that, we studied the liver, the organ most related to the pancreas, arising from the same bipotential progenitor.^39^ Carbon tetrachloride (CCl_4_) injection is a well-established model in which there is extensive free radical-induced hepatocyte death,^40^ somewhat analogous to the extensive acinar cell death induced by caerulein.

WT and PAR2KO mice were injected with a single IP dose of CCl_4_ (51.59 g/kg). While 1 day after injection, the changes in gross tissue morphology were similar (compare Supplementary Figures S19B, G, and L, with D, I, and N), the PAR2KO mice had higher biomarkers of liver damage, with higher alanine aminotransferase (ALT; Supplementary Figure S19S) and bile acid (BA; Supplementary Figure S19T) levels. As found previously, CCl_4_ led to significantly lower levels of gamma-glutamyl transferase (GGT; Supplementary Figure S19U).^41^ Seven days after CCl_4_ injection, these markers returned to normal in the WT and PAR2KO mice (Supplementary Figures S19S-U), but PAR2KO mice exhibited focal areas of hepatic necrosis (compare Supplementary Figures S19C, H, and M with E, J, and O). This could have been due to increased cell death or to a failure of regeneration. CCl_4_ did not induce apoptosis in either background as determined by cleaved caspase 3 immunostaining (Supplementary Figures S19K-O), but the PAR2KO demonstrated a failure of hepatic cells to proliferate following CCl_4_ as measured by Ki67, consistent with a defect in regeneration, whereas proliferation was evident in WT mice at 1 and 7 days following CCl_4_ (compare Supplementary Figures S19L, M with N, O, quantified in Supplementary Figure S20A).

PAR2 was barely detectable by immunohistochemistry in the normal liver (Supplementary Figure S19P), but it was markedly upregulated one day after CCl_4_ administration (Supplementary Figure S19Q). By day 7, PAR2 expression had returned almost to baseline (Supplementary Figure S19R, quantified in Supplementary Figure S20B). To determine whether the PAR2 upregulation was relevant to human liver disease, we examined data from a study of alcoholic hepatitis in humans ^42^(Supplementary Figure S1C). Similar to CCl_4_, alcohol causes hepatocellular damage through acetaldehyde, a free radical mediated mechanism.^43^ Human *F2RL1* (PAR2) mRNA was upregulated in patients with alcoholic hepatitis while *F2R* (PAR1) was not affected. PAR3 was slightly decreased (Supplementary Figure S1C). Thus, although PAR2 was not detectable in normal liver, induction is common to liver injury in both mice and humans. This is in contrast to the pancreas, where PAR2 was expressed in the normal state in both exocrine and endocrine cells, but was decreased following damage.

### PAR2 was required for regeneration of the distal phalange

To determine whether PAR2 had a role in regeneration apart from closely related organs such as the pancreas and liver, we examined digit regeneration. Human and mouse digit tips can regenerate following amputation,^44^ through a process that involves nail stem cells located in the nail epithelium^45^ and fate-restricted mesenchymal progenitors,^46^ but why regeneration fails when the amputation is proximal to the nail is not well understood. We performed amputations of the distal phalanx of WT and PAR2KO hind limbs. Amputations were introduced 3 days postnatally at three different sites: the distal tip (Supplementary Figures S21A and E) in the middle of the distal phalanx but still in the nail (Supplementary Figures S21B, F, C, and G) and at the proximal end of the phalanx (Supplementary Figures S21D and H) Two weeks following amputation, the extent of regeneration was assessed. Amputations at the most distal tip regenerated equally well in WT and PAR2KO (Supplementary Figures S21A and E, respectively), and did not regenerate in either WT or PAR2KO with amputation proximal to the nail (Supplementary Figures S21D and H). However, amputation in the middle of the distal phalanx led to effective regeneration in the WT (Supplementary Figures S21B and C) but not in the PAR2KO (Supplementary Figures S21F and G, quantified in L).

To determine whether the ability to regenerate correlated with PAR2 expression, we performed immunostaining for PAR2, finding high expression in the granular layer of the epidermis, which gradually decreased in the apical matrix (Supplementary Figures S16I-M). Expression was seen in the ventral matrix of the nail plate (Supplementary Figures S16J, O, and P), in a region that corresponds to recently described nail stem cells that express cytokeratin 17,^45^ and in the hyponychium (Supplementary Figures S21O, P, and Q). Costaining with cytokeratin 17 demonstrated colocalization in that region, consistent with expression in the nail stem cells (Supplementary Figures S21R-U). The pattern of PAR2 expression was the same in the human, with expression in the ventral matrix of the nail plate (Supplementary Figures S22A and B) and in the hyponychium (Supplementary Figures S22A–C).

## Discussion

The principal finding presented here is that PAR2 has a broad role in regeneration and death. As a GPCR that is activated by proteases, which are prevalent following tissue injury, PAR2 is well suited to sense damage and to initiate a response. In islets lacking *β*-cells, PAR2 activation was necessary and sufficient to induce *α*- to *β*- to *δ*-cell transdifferentiation. PAR2KO mice exhibited a complete lack of islet cell transdifferentiation. PAR2 was highly regulated in the islet, with each cell type exhibiting a distinct pattern of expression. PAR2 expression increased in a subset of *α*-cells in T1D and following 2fLI administration, and cells coexpressing glucagon and insulin also had high PAR2 expression, suggesting that the increased PAR2 in a subset of *α*-cells may be an early event in the transdifferentiation process. Interestingly, the PAR2 agonist 2fLI induced a transient large decrease in *β*-cell and small decrease in *δ*-cell PAR2. PAR2 was also required for exocrine regeneration and *β*-cell survival in pancreatitis.

The PAR2 modulator 2fLI was specific for islet cell transdifferentiation, with no effect on replication, consistent with our previous data.^3, 5^ This indicates that separate pathways are responsible for replication. The absence of replication following 2fLI administration is consistent with the decrease in the number of *α*-cells. The absence of replication plus the fact that the number of *δ*-cells remained unchanged as neogenic *β*-cells appeared indicates that *δ*- to *β*-cell transdifferentiation did not take place to an appreciable extent. Also, the apparent restriction of *δ*- to *β*-cell transdifferentiation to early postnatal life ^47^ is not consistent with *δ*- to *β*-cell transdifferentiation in our studies, which were done in adult mice.

We suggested a model in which *α*- to *β*-cell transdifferentiation involved at least two factors, a positive factor expressed in *α*-cells and a repressive factor expressed in *β*-cells.^5^ The data presented here support that model and indicate that PAR2 is the positive factor in *α*-cells. The induction of PAX4 transcription by 2fLI is consistent with previous data with PAX4 transgenic mice demonstrating that PAX4 is sufficient to induce transdifferentiation ^33^ and provides a mechanistic pathway for the effect of PAR2 on transdifferentiation.

Caerulein administration to PAR2KO mice led to profound and selective *β*-cell death. Selective *β*-cell loss is a cardinal feature of T1D, and also occurs in T2D. The finding that PAR2 was selectively downregulated in *β*-cells of prediabetic NOD mice provides support for the hypothesis that PAR2 is a key mediator of *β*-cell death in T1D. The mechanism by which autoimmunity and lipotoxicity induce *β*-cell death without affecting *α*- or *δ*-cells, which are related in terms of location, development, and gene expression, is not understood. To our knowledge, this is the first example of a single gene capable to mediate selective *β*-cell death in response to an environmental trigger.

The finding that PAR2 participated in regeneration in the pancreas prompted us to examine its function in other tissues, finding that it was essential to regeneration in the liver. PAR2 expression in the nail stem cells is notable. The localization of PAR2 to that region as well as the absence of regeneration of the distal phalange in the PAR2KO suggests that PAR2 is both an effector and marker of tissue regeneration.

PAR2 function appears to be limited to adult tissue homeostasis, as evidenced by the relative lack of phenotype of the homozygous *F2RL1* mutant mouse. This has implications for the paradigm that tissue regeneration in adults involves recapitulation of embryonic programs, which has been enormously influential in the field of tissue regeneration.^48^

In summary, we show that PAR2 is both necessary and sufficient for islet cell transdifferentiation in the absence of *β*-cells, that it is required to protect *β*-cells from apoptosis, and that it is highly modulated in human and murine T1D. Furthermore, we showed that PAR2 is essential in regeneration in the liver and digit. The demonstration of these roles for PAR2 in tissue regeneration provides a pharmacologically relevant target in a number of disease states.

## Materials and Methods

### Mice

C57/BL6, PAR2KO (B6.Cg-*F2rl1*^*tm1Mslb*^/J Jackson labs # 004993, Bar Harbor, ME, USA), and NOD mice were bred as homozygotes at the Sanford Burnham Prebys Medical Discovery, La Jolla, CA, USA. Glu-mT/mG mice were created as described previously^5^ with the exception that Glucagon-Cre sperm was purchased from the MMRRC (University of North Carolina, Chapel Hill, NC, USA). All animal experiments were approved by the Institutional Animal Care and Use Committee of the Sanford Burnham Prebys Medical Discovery Institute in accordance with national regulations. Males and females were used equally in all experiments.

#### Alloxan and 2fLI

C57/BL6, PAR2KO, and Glu-mT/mG mice were injected intravenously with 110 mg/kg alloxan (Sigma-Aldrich, St. Louis, MO, USA) in phosphate-buffered saline (PBS) at day 0. Mice with a blood glucose level >400 mg/dl (OneTouch Ultra Mini, Johnson & Johnson, Milpitas, CA, USA) were randomized. One group was injected with 0.1 mM/kg 2fLI (Santa Cruz Biotechnology, Santa Cruz, CA, USA) six IP injections on days 2, 4, and 6). Mice (Alloxan +/−2fLI) were killed on days 2, 6, 9, 16, and 23:

Number of mice:

Day 2 – alloxan+vehicle, *n*=4 C57/BL6 and *n*=4 PAR2KO.

Day 6 – alloxan -2fLI, *n*=6 and *n*=3 PAR2KO; alloxan+2fLI, *n*=6 and *n*=3 PAR2KO.

Day 9 – alloxan -2fLI, *n*=10, *n*=4 PAR2KO, and *n*=3 Glu-mT/mG; alloxan+2fLI, *n*=10, *n*=4 PAR2KO, and *n*=3 Glu-mT/mG.

Day 16 – alloxan +2fLI, *n*=7, *n*=4 PAR2KO; alloxan -2fLI, *n*=7 and *n*=4 PAR2KO.

Day 23 – alloxan +2fLI, *n*=4, and *n*=3 Glu-mT/mG; alloxan -2fLI, *n*=4, and *n*=3 Glu-mT/mG.

Note that three C57/BL6 mice from each group at days 9 and 16 had BrdU (1 mg/ml, Sigma-Aldrich) added to the drinking water and that three C57/BL6 mice from each group at days 6 and 9 were taken for partial islet isolation (see below).

#### 2fLI

Sixteen C57/BL6 mice were injected with 0.1 mM/Kg 2fLI (Santa Cruz Biotechnology) with six IP injections. Four mice were killed before injections, four mice immediately after 2fLI injections, four mice 1 day after 2fLI injections, and four mice 2 days after 2fLI injections.

#### Caerulein

Sixteen C57/BL6 and 20 PAR2KO mice were injected with six IP injections of 10 mg/kg caerulein in PBS (Sigma-Aldrich) on days 1, 3, and 5. Mice were monitored for survival (Supplementary Figure S16I). Mice that survived were killed on days 3, 5, and 18 (*n*=4 for each group).

#### Pancreatic damage models

Pancreas sections from NOD mice^5^ and mice that had undergone PDL^3^ were analyzed by immunostaining

#### Carbon tetrachloride

Eight C57/BL6 and eight PAR2KO mice were injected with a single IP injection of 1 ml/kg (51.59 g/kg) CCl_4_ (10% solution in olive oil). Four mice from each group were killed 1 day and 7 days after injection. Livers were harvested and analyzed.

#### Digit amputation

Three C57/BL6 and three PAR2KO mice underwent amputation at postnatal day 3. Three hind limb digits were amputated at varying sites in the distal phalanges, with the contralateral hind limb served as control. Skeletal preparations were prepared as described^49^ at postnatal day 17, 14 days after amputation. Histological sections were prepared by fixation in Zn/formalin and then embedded in paraffin.

### Human tissue

Human pancreatic tissue was obtained from the Network for Pancreatic Organ Donors with Diabetes (nPOD), a collaborative T1D research project sponsored by JDRF. Organ Procurement Organizations (OPO) partnering with nPOD to provide research resources are listed at http://www.jdrfnpod.org/our-partners.php. Information on the patients is in Supplementary Table S1. Use of that tissue was approved by the SBMRI IRB (IRB-2013-019-13). Original images from nPOD samples are available from the nPOD at http://www.jdrfnpod.org/online-pathology.php. Human finger was obtained from the National Disease Research Interchange (NDRI).

### Immunohistochemical staining

Tissue was fixed in 4% paraformaldehyde overnight at 4 °C (USB, Cleveland, OH, USA), washed in PBS, followed by overnight in 30% sucrose at 4 °C, then embedded in optimal cutting temperature, Sakura Finetek, Torrance, CA, USA) and frozen at −80 °C. Slides of 5 *μ*m thickness were washed four times with PBS and treated with 0.3% Triton in PBS for 10 min. Antigen retrieval (when applicable) was done using with CitriSolvTM (Fisher Scientific, Waltham, MA, USA) for 7 or 10 min in sub-boiling temperature. After washing with PBS for 10 min, slides were incubated in blocking solution with 5% normal donkey serum (Jackson ImmunoResearch, West Grove, PA, USA) for 60 min at room temperature. Human slides were fixed in 4% paraformaldehyde for 20 min at 4 °C, whereas all cryosections (5 *μ*m thickness) were incubated with antisera specific for PAR2 (1/400, goat, sc-8207, Santa Cruz Biotechnology), insulin (1/200, guinea pig, I7660-20B, USBIO, Swampscott, MA, USA), insulin (1/200, rabbit, sc-9168, Santa Cruz Biotechnology), glucagon (1/2000, mouse, G2654, Sigma-Aldrich), somatostatin (1/200, goat, sc-7819, Santa Cruz Biotechnology), PDX1 (1/2,000, goat, ab47383-100, Abcam, Cambridge, MA, USA), Nkx6.1 (1/1,000, mouse, AB2022, Beta Cell Biology Consortium, Nashville, TN, USA), 5-bromodeoxyuridine (BrdU; mouse, RPN 20AB, GE Healthcare, Piscataway, NJ, USA), *β*-catenin (1/400, mouse, sc-7963, Santa Cruz Biotechnology), Ki67 (1/400, rat, M7249, DAKO, Carpinteria, CA, USA) and cleaved caspase 3 (1/200, rabbit, 9664 S, Cell Signaling, Beverly, MA, USA), cytokeratin 17 (K17, 1/400, rabbit, SAB4501662, Sigma-Aldrich). To validate the PAR2 antibody, PAR2 peptide (sc-8207 P, Santa Cruz Biotechnology) was pre-complexed with PAR2 antibody at a 3 : 1 ratio for 16 h at 4 °C and used for immunostaining. Secondary antibodies for detection of guinea pig, rabbit, goat, rat, or mouse antibodies were labeled with: Alexa Fluor 488 (Invitrogen, Carlsbad, CA, USA), Alexa Fluor 649, Rhodamine Red, and Alexa Fluor 647 (all from Jackson ImmunoResearch Laboratories, West Grove, PA, USA). Nuclei were visualized with DAPI (40,6-diamidino-2-phenylindole, Sigma-Aldrich). eGFP and RFP (mT/mG) were identified directly as they are fluorescent proteins.

### Imaging and image analysis

All slides were scanned at a magnification of 20x using the Aperio Scanscope FL system (Aperio Technologies Inc., Vista, CA, USA). The appropriate dyes were assigned and illumination levels were calibrated using a preset procedure; the parameters were saved and applied to all slides. The acquired digital images represent whole tissue sections. Sections were evaluated for image quality. All acquired images were subsequently placed in dedicated project folders, and stored on a designated local server. Selected areas of the slides were selected for figures using Aperio Imagescope (version 12 Aperio Technologies Inc.). For analysis, slides were viewed, areas were selected and analyzed using the web-based Image Scope viewer. Slides were quantified using the ‘Area Quantification FL' algorithm (version 11 Aperio Technologies Inc.). The algorithm was optimized using a preset procedure to maximize the signal to noise ratio and the subsequent macro was saved and applied to all slides. Four slides that were 100 *μ*M apart were analyzed from each mouse.

### Quantification of islet cell transdifferentiation

Islet cell transdifferentiation was quantified by multiple metrics, including the % of cells expressing a single hormone, the % of cells coexpressing particular hormones, and the number of cells coexpressing a hormone found in one islet cell type along with markers such as transcription factors that are restricted to other islet cell types. These metrics were validated previously by others^32, 50^ and by us using genetic lineage tracing in the caerulein+alloxan model, where we found that hormone and marker coexpression occurred only in settings where genetic lineage tracing showed that transdifferentiation was occurring.^5^

### Quantification of PAR2 expression

To minimize variation because of differences between slides, all slides were processed together in the same solution at the same time. From each section 3–5 pancreatic islets were selected to reach roughly 200 cells. Untreated/healthy individuals were selected as controls. Endocrine cells were identified by the appropriate hormonal staining adjacent to a nucleus (positive DAPI). Areas were selected, and analyzed using the web-based Image Scope viewer. Slides were quantified using the ‘Area Quantification FL' algorithm (version 11, Aperio Technologies Inc.) and PAR2 expression levels were measured (with insulin and glucagon when applicable or as a single channel). The algorithm was optimized using a preset procedure and the subsequent macro was saved and applied to all slides. More than 200 cells were measured for each condition.

Confocal images were analyzed with ImageJ (NIH, Bethesda, MD, USA) for visualized colocalization (colocalization highlighter algorithm) and quantified (Mander‘s coefficient algorithm).

### Statistical analyses

Statistical analyses were performed with GraphPad Prism 6 (GraphPad Software, La Jolla, CA, USA) and open source R Bioconductor (www.bioconductor.org). Data distribution was assessed by a Kolmogorov–Smirnov nonparametric test of equality. Differences between two groups were assessed by two tailed Student's *t*-test or Mann–Whitney test. Differences among multiple groups were assessed by ANOVA or Kruskal–Wallis test followed by post-hoc analysis except for the experiments exploring the relationship between transdifferentiation and PAR2 expression in *α*-cells, where MANOVA was performed, with insulin and PAR2 expression defined as dependent variables, whereas experiments or predefined clusters were used as independent variables. Survival curves among groups were assessed by Mantel–Cox log-rank test. Null hypotheses were rejected at the 0.05 level. For all figures, *P<0.05, **P<0.01, ***P<0.001.

### Pancreatic islet isolation

Remnant islets from normal untreated (*n*=3), alloxan treated controls (*n*=9 at day 6 and *n*=3 at day 9), and 2fLI+alloxan injected (*n*=8 at day 6 and *n*=4 at day 9) mice were purified immediately 6 and 9 days after the last alloxan injection. Collagenase (3 mg/ml (Sigma-Aldrich) in HBSS (Cellgro, Manassas, VA, USA)) was injected into bile duct and inflated the pancreas, then removed, incubated pancreas at 37 °C for 15 min in a 50 r.p.m. water bath and washed three times with cold HBSS followed by centrifugation for 1 min at 1000 r.p.m. Liberated pancreatic cell clusters were harvested for RNA isolation and quantitative RT-PCR. As the islet structure was disrupted by alloxan, islet purification was only partial.

### Cell culture and chemical treatment

T6PNE cells were maintained in RPMI (5.5 mM glucose, Cellgro) supplemented with 10% fetal bovine serum (FBS, Sigma-Aldrich) and 1% penicillin/streptomycin (pen-strep, Gibco, Waltham, MA, USA) and grown in 5% CO_2_ at 37 °C. To induce E47 activity, 0.5 *μ*M tamoxifen (Sigma-Aldrich) was added to the culture media. PBS or 10 *μ*M 2fL1 (Santa Cruz) was added once per day for 4 days (*n*=4). Media was changed after 2 days with the addition of fresh tamoxifen. Photomicrographs of wells were captured by MetaMorph for Olympus (Olympus America, Center Valley, PA, USA) and analyzed with ImageJ (NIH) for intensity and localization.

### QPCR

RNA was purified using RNeasy Kits (Qiagen, Valencia, CA, USA), then converted to cDNA using the qScript cDNA SuperMix (Quanta BioSciences, Beverly, MA, USA). QPCR was conducted on cDNA corresponding to 2 *μ*g of RNA using an Opticon Real-Time System (MJ Research, Hercules, CA, USA) and QPCR SuperMix (BioPioneer, San Diego, CA, USA). All mRNA values were normalized to 18S rRNA values and are expressed as fold changes over vehicle-treated control.

### siRNA

Silencer siRNA (*F2RL1* siRNA ID#1960 (Ambion, Waltham, MA, USA), PAX4 siRNA #SR303361AL (OriGene, Rockville, MD, USA), or scrambled control siRNA (Ambion)) was administered to T6PNE cells by reversible transfection by mixing 2 *μ*l of individual siRNA (1 *μ*M stock) and 20 *μ*L of diluted (1:100 in Opti-MEM) Lipofectamine RNAi MAX (Invitrogen) per well of a 96-well plate (Thermo Fisher Scientific, Waltham, MA, USA), followed by incubation for 30 min at room temperature. T6PNE cells (4000 cells per well) diluted in 80 *μ*l of RPMI supplemented with 10% FBS and 1% pen-strep were added to the transfection mix and incubated for 48 h at 37 °C, 5% CO_2_, followed by the addition of 0.5 *μ*M tamoxifen. Transfected cells were incubated at 37 °C for an additional 48–72 h with tamoxifen with or without 2fLI and harvested for imaging, immunocytochemistry and RNA (*n*=4).

### Mammalian liver profile

Serum from experimental mice was collected and analyzed by a VetScan VS2 (Abaxis Veterinary Diagnostics, Union City, CA, USA) at the SBP animal core facility.

## Abbreviations

PAR2: protease-activated receptor-2
PARs: protease-activated receptors
GPCR: G-protein-coupled receptor
T1D: type 1 diabetes
T2D: type 2 diabetes
PDL: pancreatic duct ligation
PAR2KO: PAR2 knockout
2fLI: 2-furoyl-LIGRLO-amide trifluoroacetate salt
