# Supplementary material for: PAR2 regulates regeneration, transdifferentiation, and death
Source: Cell Death Dis. 2016 Nov 3;7(11):e2452–. doi: 10.1038/cddis.2016.357 (PMC5260873; doi:10.1038/cddis.2016.357)
Supplement: Supplementary Information [file cddis2016357x1.docx]

**Piran et al.**

**Supplementary Materials:**

Table S1, Figures S1-S22

Table S1. Information on human patients from nPOD

| Figure | nPOD ID # | age | sex | Duration with T1D (years) | C-peptide levels ng/ml |
| --- | --- | --- | --- | --- | --- |
| 9a | 6178 | 24 | F | Non Diabetic  Control | 4.55 |
| 9d | 6038 | 37 | F | 20 | 0.2 |
| 9f | 6052 | 12 | M | 1 | 0.18 |

**Figure S1.** **Expression of mRNA encoding PARs.** **A.** Expression in islets of mRNAs encoding PARs. RNA from five human islet preparations was isolated and sequenced. Transcript levels are expressed in RPKM units (reads per kilobase per million mapped reads). Data were extracted from ([*24*](#_ENREF_1)). Error bars are SD of the mean. **B.** *F2RL1* mRNA was increased in caerulein-induced pancreatitis. Total pancreatic RNA was isolated from saline (blue) and caerulein-treated (red) mice. Microarray analysis was done using Affymetrix GeneChips. Data were extracted from ([*38*](#_ENREF_2)) via the Gene Expression Omnibus (GEO) database. Error bars are SD of the mean. *; P value using Student’s t test for the difference in *F2RL1* mRNA between caerulein and control was 0.01. P values for *F2R* and *F2RL2* were not significant. F2RL3 mRNA was not detected. **C.** *F2RL1* mRNA was increased in human alcoholic hepatitis (red). RNA from fragments of liver from normal humans (blue) or patients with alcoholic hepatitis were analyzed using Affymetrix GeneChips. Data were extracted from ([*42*](#_ENREF_3)) via the Gene Expression Omnibus (GEO) database. Error bars are SD of the mean. ***; P value using Student’s t test for the difference in *F2RL1* mRNA between alcoholic hepatitis and control was 0.00004. P values for F2R and F2RL2 were not significant. F2RL3 mRNA was not detected.

**Figure S2. Additional examples of the effect of PAR2 on islet cell transdifferentiation.** WT and PAR2KO mice were injected with alloxan and 2fLI (Figure 1V and Methods). On days 2 (**A**), 9 (**B, E, H, J**), 16 (**C, F, I, K**), and 23 (**D, G**), pancreases were harvested and sections analyzed for insulin (red), glucagon (green), and somatostatin (white). Cells coexpressing insulin and glucagon (yellow) were common on day 9 following injection of 2fLI in WT (**E**) but not PAR2KO mice (**J**). As in the caerulein model ([*5*](#_ENREF_4)), there was a gradual increase in the number of δ-cells following 2fLI injection (**G**). DAPI (blue) was used to visualize nuclei. **L.** Kaplan-Meier survival plot of WT and PAR2KO mice injected with alloxan +/- 2fLI. As expected, 2fLI had no effect on the PAR2KO mouse (**J, K**). β-cell ablated WT mice treated with 2fLI had a survival advantage over untreated WT mice (p=0.0454), but that was not true for PAR2KO mice, where alloxan treatment led to reduced survival relative to WT mice (p=0.0105). No PAR2KO mice survived to day 23 (**L**). All p values were calculated by the Mantel-Cox log-rank test. Scale bar = 75μm**.**

**Figure S3. α-Cells transdifferentiate into β-cells, which continue to transdifferentiate into δ-cells.** Cells expressing glucagon and insulin (**A** – low power, and **B** – high power views) are seen 9 days after alloxan injection, which corresponds with the α- to β-cell transdifferentiation intermediate. Cells expressing insulin and somatostatin (**C** – low power, and **D** – high power views) are seen 23 days after alloxan injection, which corresponds with the β- to δ-cell transdifferentiation intermediate. No cells expressing glucagon and somatostatin were found. Pancreas sections were stained for insulin (red), somatostatin (white), glucagon (green), DAPI was used to visualize nuclei. Scale bar = 75μm for the low power view, = 7μm for the high power view.

**Figure S4. β-cell increase is due to transdifferentiation and not to apoptosis or replication.** An islet from a mouse 9 days post treatment with alloxan+2fLI. None of the ins+ cells were positive for Ki67 (green) or cleaved caspase 3 (white).

**Figure S5. mT/mG mice had no eGFP expression prior to Cre-mediated recombination.** An islet from B6.129(Cg)-*Gt(ROSA)26Sor^tm4(ACTB-tdTomato,-EGFP)Luo^*/J mouse showing that eGFP is not expressed in pancreatic islets before mating with a mouse expressing Glu-cre and very low levels of eGFP in some acinar cells. **A.** Color combined. **B.** eGFP and DAPI only.

**Figure S6. Recombination rate of Glu-mT/mG mice.** To calculate the recombination rate, the number of α-cells expressing mT or eGFP were quantified. α-cells from 10 different islets were counted and 60.05% were eGFP positive. **A**. an islet from a Glu-mT/mG mouse stained for glucagon (white). (**B, D**) High power views of eGFP+ cells expressing glucagon. (**C**) High power view of unrecombined α-cell expressing glucagon and mT. E. eGFP channel demonstrating restricution of recombination to α-cells.

**Figure S7. High power views of islets demonstrating that newly formed β-cells originated from preexisting α-cells.** (**A**) Islet from Figure 2B and (**E**) islet from Figure 2D, 9 and 23 days post alloxan+2fLI, respectively. Pink squares (**D, F**) contain eGFP negative, insulin positive cells representing preexisting β-cells, and yellow squares (**B**, **C, G, H**) contain eGFP positive, insulin positive cells, representing β-cells newly formed from α-cells. Scale bar = 75μm for the low power view, = 7μm for the high power view.

**Figure S8. High power views of islets demonstrating that newly formed δ-cells originated from preexisting α-cells.** (**A**) Islet from Figure 2H and (**E**) islet from Figure 2J, 9 and 23 days post alloxan+2fLI, respectively. Pink squares (**B, F**) contain eGFP negative, somatostatin positive cells representing preexisting δ-cells, and yellow squares (**C, D, G, H**) contain eGFP positive, somatostatin positive cells, representing δ-cells newly derived from α-cells. Scale bar = 75μm for the low power view, = 7μm for the high power view.

**Figure S9. endocrine-cell transdifferentiation is islet autonomous.** Pancreas sections from lineage-tracing mice sacrificed 23 days following alloxan plus 2fLI. **A.** An islet with no preexisting β-cells (white cytoplasmic insulin and red mT membrane fluorescence), demonstrating neogenic β-cells (white cytoplasmic insulin and green eGFP membrane fluorescence) (high power views in **B, C,** neogenic cells marked with yellow arrows). **D.** An islet with many preexisting β-cells (high power views in **E, F,** preexisting cells marked with pink arrows) demonstrating no neogenic β-cells. **G.** Quantification of neogenic β-cells per islet. The number of preexisting β-cells in each islet (insulin+, mT+) was plotted against the number of neogenic β-cells (insulin+, eGFP+) in that islet. The p-value for the difference between islets in which there were greater than 7 preexisting β-cells and at least one eGFP-positive cell expressing insulin (quadrant 2) versus islets in which there were 7 or fewer β-cells expressing mT and at least one eGFP-positive cell expressing insulin (quadrant 4) was smaller than 2e-10, calculated using a two sample proportion test with continuity correction in R. Scale bar = 75μm for the low power view, = 7μm for the high power view.

**Figure S10. 2fLI induced transdifferentiation but not replication**. BrdU was administered in the drinking water for 16 days beginning at the time of alloxan injection. 3 mice each were sacrificed 9 (**A**) and 16 (**B**) days after alloxan injection. While there was an increase in the number of cells expressing insulin (red) and somatostatin (white) over time, the newly appearing insulin and somatostatin-positive cells were negative for BrdU (green). The intestine from the same mouse as in **B** was used as a positive control for BrdU incorporation (**C**). Scale bars = 75μm.

**Figure S11. High power views of normal islets demonstrating specificity of the PAR2 antibody and differential PAR2 expression levels in islet cells. A-D.** PAR2 antibody specificity. The sections from same normal mouse pancreas, were stained with PAR2 antibody only **(A, B)** or PAR2 antibody with a blocking peptide comprising the region of the protein used to generate the antibody **(C, D)**. To demonstrate specificity of the PAR2 antibody, a, PAR2 peptide (sc-8207P) comprising the region of the protein used to generate the antibody was incubated with PAR2 (S-19, sc-8207) antibody for 16hr in 4C (PAR2 Ab-Ag). No PAR2 staining was seen in the PAR2KO mouse (not shown). **E-G.** Differential PAR2 expression in islet cells. High power views of the islet shown in Figure 4U (E), demonstrating high PAR2 expression levels (white) in δ-cells (somatostatin, red) and lower PAR2 levels (white) in α-cells (glucagon, green F, G). Scale bar = 7μm.

**Figure S12. PAR2 expression in prediabetic NOD mice.** An islet without insulitis from a pre-diabetic NOD mouse. **A.** Staining for DAPI, insulin (red), amylase (green), and PAR2 (white). **B.** Staining for DAPI, somatostatin (red), glucagon (green) and PAR2 (white). Compare to islets with insulitis in Figure 4. Scale bars=75μM.

**Figure S13. Additional example of high power views of normal islets demonstrating differential PAR2 expression levels in different islet cells. A-D.** High power views of the islet shown in Figure 6Q, demonstrating high PAR2 expression levels (white) in δ-cells (somatostatin, red) and lower PAR2 levels (white) in α-cells (glucagon, green F, G). Scale bar = 7μm.

**Figure S14. Bimodal expression of PAR2 in α-cells 1 day after 2fLI injection.** High power view of indicated regions from Figure 6S, showing α-cells (GLU, green) with high (**A, C,** yellow squares) and low (**B, D,** pink squares) expression of PAR2 (white). Note that δ-cells (SOM, red) uniformly express a high level of PAR2. DAPI (blue) was used to visualize nuclei. Scale bar=7μm.

**Figure S15. Bimodal expression of PAR2 in α-cells 2 days after 2fLI injection.** High power view of indicated region from Figure 6T, showing α-cells (GLU, green) with high (**A, D,** yellow squares) and low (**B, C,** pink squares) expression of PAR2 (white). Note that δ-cells (SOM, red) uniformly express a high level of PAR2. DAPI (blue) was used to visualize nuclei. Scale bar= 7μm.

**Figure S16. β-cell ablation did not induce increased PAR2 or insulin expression in α-cells.** Normal (**A-C**) and β-cell ablated (**E-G**) mice were analyzed for insulin (red), glucagon (green) and PAR2 (white). Low power (**A, E**) and high power (**B, C, F, G**) views. Quantification of PAR2 and insulin expression in cells that expressed glucagon from normal (**D**) and β-cell ablated (**H**) mice. β-cell ablation did not induce a significant difference in PAR2 or insulin expression. Scale bar= 7μm

**Figure S17. Differential PAR2 expression in human Type I diabetes.** Separation of the PAR2 channel from the hormones of the human islet presented in Figure 9D.

**Figure S18. Effect of 2fLI and *F2RL1* siRNA on PAR2 and insulin expression.** T6PNE cells were treated with PBS (**A, G**), 2fLI (10μM) for 4 days (**B, H**), scrambled siRNA (**C, I**), or *F2RL1* siRNA (**D, J**). **E, F**. The number of cells expressing eGFP, indicating expression of the human insulin promoter-GFP transgene introduced into T6PNE cells ([*30*](#_ENREF_5)), in response to 2fLI (**E**) or F2RL1 siRNA (**F**). **G-J.** T6PNE cells treated as in A-D were stained for PAR2 (quantified in **K, L**). Scale bars=200μm. Error bars are SEM.

**Figure 19. PAR2 modulated hepatocellular damage and regeneration following CCl_4_ injection. A-E.** Macroscopic appearance of livers (indicated by dashed white line) from WT (**A-C**) and PAR2KO (**D, E**). **F-J**. Low-power view stained with hematoxylin and eosin, with a high-power view of the indicated region below. **K-O**. Cleaved caspase 3 (red) and Ki67 (green) immunostaining. A positive control for Ki67 and cleaved caspase 3 staining is in Figure 12J, K). Arrows point to cells with turquoise nuclei that have nuclear Ki67 staining (confluence of green Ki67 and blue DAPI staining). Because of the large amount of necrotic tissue, particularly at the 1 day time point, there is nonspecific staining (yellowish from red and green colocalization). **P-R.** PAR2 immunostaining (brown, quantified in Figure S14B). **S-U.** Biomarkers of liver damage following CCl_4_ injection. **S**. Alanine aminotransferase (ALT) Note that the upper limit of the assay is 2,000 μM. **T**. Bile acid (BA). **U**. Gamma glutamyl transferase (GGT). Black asterisks mark statistically significant differences between the two time-points in the same experimental group, while red asterisks mark statistically significant differences between WT and PAR2KO mice 1 day after injection. Error bars are SEM. Scale bars= 75μM.

**Figure S20. Quantification of proliferation and PAR2 expression following CCl_4_ administration. A.** Quantification of replication following liver injury in the PAR2KO mouse. Sections from livers of WT and PAR2KO mice were stained with Ki67 antibody and the number of positive nuclei per 100 μm^2^ of liver tissue was counted. **B.** Quantification of PAR2 expression over time following CCl_4_ administration in WT mice.

**Figure S21. PAR2 is required for regeneration of the distal phalanx.**

**A–I.** Skeletal preparations at postnatal day 17 stained for bone with Alizarin Red and for cartilage with Alcian Blue. For each set of three panels, pictures were taken with dorsal (left) lateral (middle), and a magnification of the lateral view (right). Black dotted lines surround the WT distal phalange, where regeneration of a properly shaped nail tip occurred, while blue dashed lines surround the PAR2KO distal phalange which exhibited cone shaped areas of poorly regenerated tissue. **A, E**. distal tip amputation: WT and PAR2KO regenerate suitable distal tips **B, C, F, G**. Amputation was performed in the middle of the distal phalanx within the nail (2 examples). **D, H**. Amputation in both WT and PAR2KO was performed at the proximal end of the distal phalange, proximal to the nail bed. No regeneration was observed under that condition. **I.** The area of the distal phalange was measured using ImageJ, demonstrating a significant difference (p=0.028) between WT and PAR2KO. All amputations were performed at postnatal day 3 and analyzed at postnatal day 17. **J-N.** PAR2 immunohistochemistry showing low power view of the entire hindlimb (**J**) and higher power views of the indicated regions (**K-N**). **O-Q**. PAR2 expression (brown) in the region of the distal phalange containing the nail at low (O) and high (P, Q) power. superimposed with regions reported to express keratin 17 (K17), a marker of nail stem cells (45).. **R-U**. Colocalization of PAR2 (green) and K17 (red) at low power (**R**) and high power (**S-U**). Nuclei were visualized with DAPI (blue). Scale bars= 75μM.

**Figure S22. PAR2 expression in the human finger is similar to that of the mouse.** PAR2 immunohistochemistry (Brown-orange) showing low power view of the dorsal section of the human distal phalanx (**A**) and higher power views of the indicated regions (**B, C**). Note that the section shown in **B** near the nail fold corresponds with the section shown in Figure S21P and the section shown in **C** containing the hyponychium (arrow) corresponds with the section shown in Figure S21Q. Scale bars= 1.7mm.
