# Supplementary figures and images for: PAR2 regulates regeneration, transdifferentiation, and death
Source: Cell Death Dis. 2016 Nov 3;7(11):e2452–. doi: 10.1038/cddis.2016.357 (PMC5260873; doi:10.1038/cddis.2016.357)

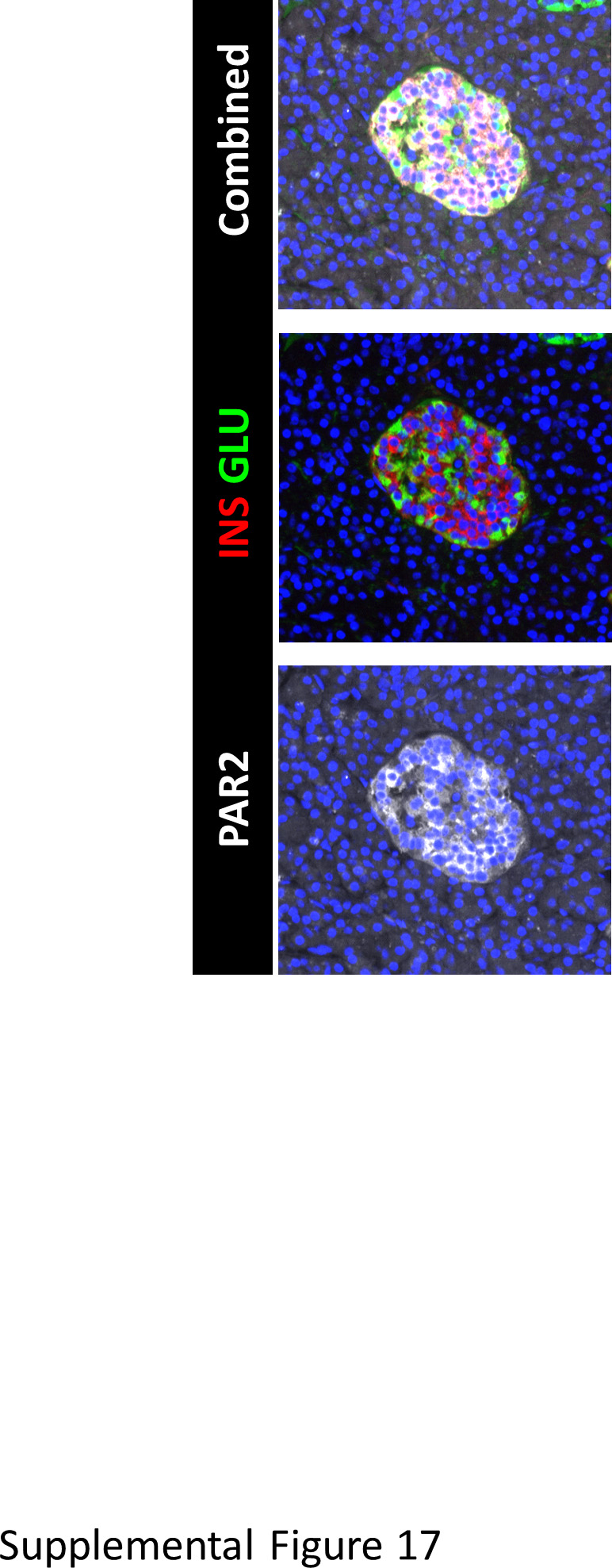

Supplement: Supplementary Figure 17 [file cddis2016357x18.tif]
